# Meta-regression with categorical moderators and dependent effect sizes: A simulation study

**DOI:** 10.1017/rsm.2026.10092

**Published:** 2026-05-14

**Authors:** Belén Fernández-Castilla, José Antonio López-López, María Rubio-Aparicio

**Affiliations:** 1 Department of Methodology of Health and Behavioral Sciences, Faculty of Psychology, Universidad Nacional de Educación a Distancia, Spain; 2 Basic Psychology & Methodology, University of Murcia, Spain; 3 IMIB-Arrixaca, Murcian Institute of Biomedical Research, Spain

**Keywords:** dependent effect sizesmultilevel modelspowerrobust variance estimationsimulationType I error

## Abstract

Categorical moderators are often found in meta-analysis and examined using meta-regression models. When multiple effect sizes are present within studies, several methods can be used for meta-regression: multivariate models, three-level models, correlated-effects models with robust variance estimation (RVE), three-level models with RVE, and correlated-effects models with RVE and cluster wild bootstrapping (CWB). This study aimed to compare the performance of these methods through a simulation study. Cohen’s *d* values were generated under a multivariate model, incorporating a binary variable that could represent either study-level or effect size-level characteristics. When the moderator referred to an effect size-level characteristic, its effect was allowed to vary across studies. Factors manipulated in the simulation included number of studies, number of outcomes per study, and the distribution of effect sizes across the categories of the moderator variable, ranging from balanced to highly unbalanced. The methods were applied and compared in terms of bias, Type I error, and power. The results showed that all methods exhibited lower power to detect effects when the moderator variable referred to study-level characteristics and the effect size distribution was very unbalanced. Methods based on RVE (correlated-effects with RVE or with RVE and CWB, and three-level models with RVE) effectively controlled Type I error rates but tended to be overconservative. In contrast, three-level models achieved higher power but at the cost of inflated Type I error. The best balance between Type I error control and power was observed when using a combination of three-level models and RVE.

## Highlights

### What is already known?

- Moderator analyses are a crucial step in a meta-analysis, although they are often underpowered.
- In the presence of multiple effect sizes within studies, various methods are available for analyzing moderator variables through meta-regression, including multivariate models, multilevel models, robust variance estimation, and combinations of the first two methods with robust variance estimation.
- Some simulation studies have compared the performance of these methods. The results indicate that all of them achieve adequate Type I error rates with a large number of studies and that a posterior robust variance correction addresses model mis-specification.
- Despite this, some questions remain: The statistical power of these methods has never been directly compared, nor have Type I error rates or power been evaluated when the moderator variable pertains to study characteristics.

### What is new?

- This article provides a comprehensive comparison of the statistical properties of methods for conducting meta-regressions when multiple effect sizes are present within studies and binary variables are analyzed, related to either study-level or within-study characteristics.
- We focus on a common scenario in this context: studies or effect sizes are often highly imbalanced across the categories of the categorical moderator variable.
- The results show that the properties of these methods vary depending on whether moderator variables refer to study-level or effect size-level characteristics: Statistical power is negatively affected by the imbalanced distribution of effect sizes, and it is generally higher when the moderator variable pertains to within-study characteristics.
- Multilevel models alone can lead to inflated Type I error rates when moderator variables refer to within-study characteristics, but this inflation may be corrected using robust variance estimation.

### Potential impact for *RSM* readers

- The results of this study will help applied meta-analysts select the most suitable methods for conducting meta-regression.

In meta-analysis, findings from studies investigating the same research question are aggregated quantitatively to provide a comprehensive overview of the available evidence pertaining to that specific topic. The unit of analysis is typically an effect size, which is a number that summarizes the strength of the association between the variables of interest. Following the compilation of this evidence summary, the next step commonly involves checking whether there is statistical heterogeneity among the effect sizes, and if that is the case, exploring whether the characteristics of the studies have an impact on the observed effect sizes.^1^ For example, in Yan and colleagues’ meta-analysis^2^ examining the influence of peer assessment on academic outcomes, the researchers explored whether studies that incorporated online technology in their procedures yielded larger effect sizes compared to those that did not. Their findings revealed that, indeed, the overall effect size was greater for the former (*g* = 0.78) than for the latter (*g* = 0.44). This procedure of using study characteristics to predict study results is known as moderator analyses and is commonly carried out through the implementation of a mixed-effects model, also known as meta-regression.^3^

Moderator analyses play an important role in advancing scientific understanding by uncovering how different study characteristics might affect the results obtained. These analyses are crucial because they direct researchers’ attention to factors that might have been previously overlooked. When significant moderator effects are observed, these previously ignored factors become salient, leading to a more refined picture of the phenomena being studied. This process enriches the overall understanding and interpretation of research findings, ultimately contributing to more robust and comprehensive scientific insights.

Moderator analyses are commonly conducted through statistical regression models known as meta-regressions. In these models, a study characteristic either categorical (e.g., type of design) or quantitative (e.g., mean age of the sample) is used as a predictor variable in a regression where the dependent variables are the effect sizes extracted from primary studies. Given the important role of moderator analyses in scientific advancement, it is crucial to ensure that the estimates derived from meta-regression models are unbiased, effectively control Type I errors, and maintain robust statistical power to detect an effect.

Numerous studies have focused on these aspects of meta-regression analysis in situations where only one effect size could be extracted per study.^4^ ^–^ ^13^ However, studies incorporated into meta-analyses often report multiple results, frequently supplying data that allows the calculation of several effect sizes.^14^ For example, when different questionnaires are used to measure different aspects of a psychological construct (e.g., to measure anxiety, different facets of the construct can be measured, such as motor, cognitive, or physiological anxiety), measurements are made on different subsamples, or the same sample is measured at different time points. These effect sizes are more closely related to each other because they stem from the same study and therefore share underlying methodological characteristics, or because they are derived from the same individuals, making them statistically dependent. This dependency must be considered in the statistical model of the meta-analysis to avoid a Type I error rate higher than the nominal value.^15^

Three primary methods are used to model the dependence between effect sizes: the multivariate method,^16^ ^,^ ^17^ three-level models,^18^ ^,^ ^19^ and correlated-effects models using the robust variance estimation (RVE) inference method.^20^ ^,^ ^21^ Recently, a combination of three-level models with RVE method has been proposed to ensure nominal Type I errors.^22^ ^–^ ^25^ Last, to enhance the power to detect moderator effects, a new inferential technique called cluster wild bootstrapping (CWB) has been proposed within the correlated-effects model with RVE framework.^26^ The technical details of these methods will be introduced in the upcoming sections.

All these methods can accommodate moderator variables to explain the variability observed in effect sizes both across studies and within studies. Moderator variables can reflect study characteristics (e.g., the country where the study is conducted) or within-study characteristics (e.g., instruments used to measure multiple dependent variables reported within studies). Throughout this text, we will refer to these two types of characteristics as study-level variables and effect size-level variables, respectively. Although not addressed in the present manuscript, it is important to consider centering these moderator variables (both continuous and categorical) to disentangle level-specific effects.^27^ ^,^ ^1^

Several studies have investigated or compared the effectiveness of the aforementioned methods in the context of meta-regression, examining both quantitative and categorical study-level and effect size-level characteristics.^9^ ^,^ ^21^ ^,^ ^22^ ^,^ ^26^ ^,^ ^28^ López-López et al.^9^ compared the power and Type I error of three-level models and correlated-effects models with RVE inference method in meta-regressions with a single quantitative moderator variable that could describe a study or an effect size characteristic. Results showed that the three-level multilevel model had higher power but poorer control of Type I error, while the correlated-effects models with RVE method provided correct Type I error rates but had low statistical power. Unfortunately, this research focused exclusively on quantitative moderator variables, whereas many times moderator variables are categorical, such as the educational level of the sample (primary, secondary, and university) or the type of design (experimental, quasi-experimental, and observational).

Regarding the performance of meta-regressions with categorical variables, Tipton^21^ found that applying the RVE inference method to a correlated-effects model with different small-sample adjustments, such as the bias-reduced linearization correction proposed by Bell and McCaffrey^29^ combined with the Satterthwaite approximation for estimating degrees of freedom,^30^ provided the best tradeoff between Type I error and statistical power when analyzing unbalanced dichotomous variables. This was particularly true when the number of studies was 40, the between-study heterogeneity was small or moderate, and the effect size difference between categories was large (i.e., 0.40). However, in other simulated conditions, statistical power was very low. A limitation of this study is that the performance of the correlated-effects model with RVE and small-sample adjustments was not compared to other methods, such as three-level models or three-level models with RVE.

This limitation is also present in the simulation study conducted by Joshi et al.,^26^ who examined the Type I error and power of the CWB method applied to a correlated-effects model with RVE using various types of variables (i.e., quantitative and categorical, both at study and at effect size level). This method is very promising, as it was found to adequately control Type I error while showing higher statistical power than the correlated-effects model with RVE and small-sample correction, particularly when hypothesis tests involved multiple contrasts, with as few as 10 studies. However, the authors did not compare the performance of CWB with other alternative methods.

Two existing simulation studies have compared the performance of the three-level model and the correlated-effects model with RVE inference in meta-regressions with categorical moderator variables.^22^ ^,^ ^28^ Park and Beretvas^28^ found that the three-level model had convergence issues and underestimated standard errors under unfavorable conditions (i.e., a small number of studies, high heterogeneity, and an uneven distribution of effect sizes across moderator categories). In contrast, the correlated-effects model with RVE showed better performance. In a subsequent study, Fernández-Castilla et al.^22^ extended this research by incorporating additional simulation conditions, testing alternative three-level model specifications (i.e., a three-level model that allows estimating different between-studies residual variance estimates for each category of the moderator) and evaluating hybrid approaches that combined three-level models with RVE. They concluded that all methods provided adequate estimates when at least 40 studies are available. Additionally, they found that applying three-level models with RVE generally maintained appropriate Type I error rates, even when the three-level model was mis-specified and the number of studies was as low as 20. Lastly, they observed that an imbalance in the number of effect sizes across categorical moderator categories negatively impacted results.

Despite the advancements made by these studies, a critical gap remains. Neither of these studies examined or compared the statistical power and Type I error rates^2^ of the methods for detecting differences in overall effects across categorical moderator categories. Additionally, both studies focused on moderator variables that represented effect size-level characteristics, but not on study-level characteristics. Addressing these gaps is the primary motivation for the present study.

## 1 The present study

Categorical variables are of special interest from a methodological perspective because, in meta-analysis, effect sizes are often disproportionately distributed across categories (see example provided in Table 1). This imbalanced distribution is known to affect the properties of methods commonly used in meta-regressions.^21^ ^,^ ^22^ Beyond the type of variable used in a meta-regression, when a variable describes an effect size-level characteristic, different three-level model specifications can be applied, and choosing an incorrect specification can significantly underestimate standard errors associated with the moderator effect,^22^ leading to inflated Type I error rates.

**Table 1:** Results from meta-regressions carried out with different methods Table 1 Long description.

	Estimates (SE)	*p*-Value	Variance estimates
Multivariate method			
Intercept	0.374 (0.065)		${\hat{\sigma }}_{u_{res}}^{2}$ **=** 0.330
Difference	0.413 (0.079)	< 0.001	
Three-level model			
Intercept	0.518 (0.076)		${\hat{\sigma }}_{u_{res}}^{2}$ = 0.390 ${\hat{\sigma }}_{w_{res}}^{2}$ = 0.080
Difference	0.219 (0.133)	0.099	
Three-level with additional random effect			
Intercept	0.535 (0.074)		${\hat{\sigma }}_{w_{res}}^{2}$ = 0.075
Difference	0.177 (0.161)	0.271	${\hat{\sigma }}_{u_{res}0}^{2}$ (76) = 0.358 ${\hat{\sigma }}_{u_{res}1}^{2}$ (26) = 0.550
Three-level model with RVE			
Intercept	0.518 (0.074)		${\hat{\sigma }}_{u_{res}}^{2}$ = 0.390
Difference	0.219 (0.167)	0.204	${\hat{\sigma }}_{w_{res}}^{2}$ = 0.080
Correlated-effects models with RVE			
Intercept	0.550 (0.076)		${\hat{\sigma }}_{u_{res}}^{2}$ **=** 0.304
Difference	0.044 (0.152)	0.772	
Correlated-effects models with RVE and CWB			
Intercept	*–*		
Difference	*–*	0.782	

*Note:* ${\hat{\sigma }}_{u_{res}}^{2}$ = residual between-studies variance; ${\hat{\sigma }}_{w_{res}}^{2}$ = residual within-study variance; RVE = robust variance estimation; CWB = cluster wild bootstrapping; SE = standard error. To fit the multivariate model, an imputed variance–covariance matrix was used, using a correlation of 0.6.

To date, no study has conducted an integrative analysis comparing Type I error rates and statistical power across various methods (and their specifications) for meta-regression with binary variables, which can represent study- or effect size-level characteristics, in the presence of dependent effect sizes. This research gap serves as the primary motivation for the present simulation study. The results of this study will help meta-analysts determine which method exhibits better properties under different conditions when analyzing categorical moderator variables. Additionally, it will enable them to make more informed decisions regarding the inclusion (or exclusion) of meta-regression analyses involving binary variables with a high imbalance across categories. Finally, this article aims to clarify the different three-level model specifications that can be implemented when effect size-level moderators are present, along with their respective pros and cons.

## 2 Methods for meta-regression

In this section, we introduce the meta-regression models that will be tested in the present simulation study: three-level models, correlated-effects model with RVE inference, their combination (three-level models with RVE), and correlated-effects models with RVE and CWB as inferential methods. For clarity, we will explain the different terms in the equations using an applied example. Yan et al.^2^ published a meta-analysis on the effect of peer assessment (compared to teacher assessment) on academic outcomes. A relevant (binary) moderator variable was whether students received feedback from their peer assessments (coded as 0 – No and 1 – Yes).

### 2.1 Three-level models

A traditional random-effects model can be seen as a two-level model, where effect sizes are nested within studies. In the presence of multiple effect sizes, an additional level (or random effect) can be added to account for the dependency among these effects, which are often extracted from the same sample or following identical procedures.

The three-level model employed to estimate an overall effect size as well as the variability within and across studies, along with its advantages and disadvantages, has been thoroughly described in previous manuscripts.^18^ ^,^ ^19^ ^,^ ^31^ In general, the primary advantage of this approach is that it does not require knowledge of the covariances among effect sizes, unlike the multivariate approach. Additionally, it treats different outcomes (i.e., various effect sizes within studies) as a random sample from a population of outcomes, rather than a fixed set of outcomes identical from study to study, which might not be realistic in many contexts.

In the following lines, we delineate two types of three-level mixed-effects models applicable for conducting moderator analyses. Initially, we elaborate on the mixed-effects model integrating a binary moderator variable denoting a characteristic of the studies. Subsequently, we discuss a mixed-effects model encompassing a categorical moderator variable reflecting characteristics of the outcomes within studies (e.g., at the effect size level). These models are expounded separately due to the latter’s potential specification in two distinct manners, but in practice, it is possible to incorporate moderator variables at both levels at the same time.

*Study-level characteristics.* When a meta-analyst aims to test the impact of study characteristics on the observed effect sizes, they can implement the following three-level model. At Level 1, the term $t_{ij}$ refers to the *i*th effect size observed in study *j*:

(1) $$\begin{array}{r} t_{ij}=\beta_{ij}+e_{ij}.\;\left(\mathrm{Level}\;1\right) \end{array}$$

Following the example introduced before, $t_{ij}$ would be the standardized mean difference that indicates the difference between the group that received peer assessment and the group that received teacher assessment in academic performance, measured with outcome *i* (e.g., math exam, science exam, English assignment, etc.) in study *j.* This observed effect size is not exactly equal to its population effect, $\beta_{ij}$ , due to a random deviation, $e_{ij}$ , that emerges due to the use of different samples across outcomes^3^ and studies. These deviations are assumed to be normally distributed with mean 0 and variance $\sigma_{e_{ij}}^{2}$ , typically known as the sampling variance, which is often estimated before conducting the meta-analysis.

At Level 2, the population effect $\beta_{ij}$ equals the population mean for study *j*, $\theta_{0j}$ , plus a random deviation, $w_{ij}$ , whose variance ( $\sigma_{w}^{2}$ ) would be interpreted as the within-study variance:

(2) $$\begin{array}{r} \beta_{ij}=\theta_{0j}+w_{ij}.\;\left(\mathrm{Level}\;2\right) \end{array}$$

Following the example, the population effect size reflecting the difference between the peer-assessment group and the teacher-assessment group in outcome *i* within study *j (* $\beta_{ij}$ *)* equals the mean of the population effect of study *j*, $\theta_{0j}$ , plus a random deviation $w_{ij}$ that emerges due to the fact that the outcomes used to measure academic performance may differ within studies: maybe authors used both a math and a reading assignment. The variance of $w_{ij}$ , $\sigma_{w}^{2}$ , reflects the variability in the population effects around the study-specific mean $\theta_{0j},$ that is, the within-study variance.

Lastly, at Level 3, the mean population effect for study *j*, $\theta_{0j}$ , may vary either randomly or in relation to the characteristics of the study:

(3) $$\begin{array}{r} \theta_{0j}=\gamma_{00}+\gamma_{01}Z_{1j}+u_{0j}.\;\left(\mathrm{Level}\;3\right) \end{array}$$

The term $Z_{1j}$ of Equation (3) refers to any characteristic of study *j*. If this characteristic is categorical such as study design (e.g., experimental or quasi-experimental), or whether the students received feedback from their assessments (e.g., yes or no), the term $\gamma_{00}$ would refer to the overall effect size for the reference category. For instance, if the “feedback” variable was dummy coded in 1s and 0s, $\gamma_{00}$ would represent the overall standard mean difference for those studies where the students did not receive feedback on their assessments. The term $\gamma_{01}$ would represent the difference between the pooled standardized mean differences of both categories, and therefore, $\gamma_{00}$ + $\gamma_{01}$ would equal the pooled standardized mean difference for those studies where the students did receive feedback of their assessments. Finally, the random effect $u_{0j}$ , assumed to be normally distributed with mean 0 and variance $\sigma_{u_{res}}^{2}$ , is the residual between-studies variance after accounting for the effect of $Z_{1j}$ .

*Effect size-level characteristics.* Moderator variables can also pertain to characteristics of effect sizes within studies. For example, if some studies include two conditions: one where feedback was not provided to students and another where feedback was provided, different effect sizes could emerge within a study depending on the presence or absence of feedback. Therefore, this variable now describes an effect size-level characteristic. If this categorical moderator was analyzed with a three-level meta-regression model, the first level (Equation (1)) would remain the same. However, at Level 2, the population outcome-specific effect $\beta_{ij}$ would exhibit variability, stemming from both random factors and the attributes of the outcomes:

(4) $$\begin{array}{r} \beta_{ij}=\theta_{0j}+\theta_{1j}X_{1ij}+w_{ij}.\;\left(\mathrm{Level}\;2\right) \end{array}$$

The term $X_{1ij}$ represents a characteristic of outcome *i* within study *j* (e.g., absence or presence of feedback), and the term $\theta_{1j}$ is the effect of $X_{1ij}$ on the population outcome effects $\beta_{ij}$ within study *j.* Importantly, at Level 3, we can make two assumptions regarding the within-study effect $\theta_{1j}$ : either it remains constant across studies (e.g., the presence of feedback systematically improves or worsens the assessment of academic performance to the same extent across studies) or it varies across studies (e.g., the presence of feedback on academic performance differs between studies). In practice, meta-analysts often apply the first model specification, where at the study level (Level 3), the effect $\theta_{1j}$ remains constant across studies ( $\gamma_{10}$ ):

(5) $$\begin{array}{r} \{{}_{\theta_{1j}=\gamma_{10}}^{\theta_{0j}=\gamma_{00}+u_{0j}}\;\left(\mathrm{Level}\;3\right). \end{array}$$

The term $\gamma_{00}$ represents the overall effect size in the reference category. Following our example, $\gamma_{00}$ would denote the overall standardized mean difference for those outcomes where feedback was absent. Unlike the moderator effect $\gamma_{10}$ , the effect $\gamma_{00}$ is typically allowed to vary across studies (the random effect $u_{0j}$ is assumed to be normally distributed with mean 0 and variance $\sigma_{u_{0}}^{2}$ , which is the between-studies variance of the overall effect size for the reference category). An important implication of the model is precisely the assumption that the effect $\gamma_{10}$ (i.e., the difference between the overall effect across categories) is the same or stable across studies. If $\gamma_{10}$ did vary across studies, that is, if the effect of feedback on academic performance differed between studies, the model in Equation (5) would be mis-specified, and that can have important implications on the parameter estimates.^22^ Alternatively, $\gamma_{10}$ could be allowed to vary across studies:

(6) $$\begin{array}{r} \{{}_{\theta_{1j}=\gamma_{10}+u_{1j}}^{\theta_{0j}=\gamma_{00}+u_{0j}}\;\mathrm{Level}\;3. \end{array}$$

Under this model, the moderator effect of variable $X_{1ij}$ on the outcome effects within study *j* ( $\theta_{1j})$ would equal an overall moderator effect, $\gamma_{10}$ , plus a random deviation, $u_{1j}.$ The variance of $u_{1j}$ is $\sigma_{u_{1}}^{2}$ , which would represent the heterogeneity of $\gamma_{10}$ across studies.

To assess the statistical significance of the moderator effect ( $\gamma_{01}$ or $\gamma_{10}$ ) which represents the difference between categories, Wald tests are commonly employed, with a significance level of 0.05. Alternatively, likelihood ratio tests (LRTs) can be used to compare the fit of a null model to that of a model incorporating the moderator variable. If the model with the moderator variable shows a significantly better fit ( α = 0.05), it can be concluded that the moderator variable is statistically significant.

Recently, a method using three-level models in combination with RVE inference method has been proposed. This method consists of applying first a three-level meta-regression, then extracting the variance–covariance matrix from the three-level model results, and then correcting this matrix using a sandwich estimator, including the bias-reduced linearization adjustment of Bell and McCaffrey.^29^ Also, hypothesis tests using Satterthwaite degrees of freedom are conducted. This posterior application of RVE ensures that inferences are correct in terms of Type I error, even if the three-level model is mis-specified.^22^ ^,^ ^24^

### 2.2 Correlated-effects model with RVE inference

This method involves the specification of a working model that accounts for the correlation between effect sizes within studies. It builds on the random-effects model but includes a sandwich-type adjustment to the standard errors of the regression coefficients. This adjustment helps to prevent underestimation of standard errors due to statistical dependence among effect sizes.^20^ A great advantage of this method is that there is no need to estimate covariances between effect sizes, because it relies on a working model that involves making general, approximate assumptions about the dependence structure. Additionally, this method does not make distribution assumptions, as opposed to the three-level model which requires the sampling distribution of the effect sizes to be normal.

The statistical model for meta-regression is the following:
(7)

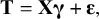

where T is a stacked vector containing the *i* effect sizes within the *m* studies, and **X** is a design matrix constructed so that its rows correspond to the total number of effect sizes, while its columns represent the number of covariates examined in the meta-regression analysis (plus the intercept, if desired). These covariates can refer to characteristics of the studies or of the outcomes. Moreover, γ represents a vector containing the regression coefficients (i.e., moderator effects) that need to be estimated, and 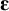 is a stacked vector with the residuals of each of the *k* effect sizes within the *m* studies. To estimate the vector γ of regression coefficients, weighted least squares estimation method is used:

(8) $$\begin{array}{r} \hat{\text{γ}}={\left(\sum_{j=1}^{m}X_{j}^{\prime }W_{j}X_{j}\right)}^{-1}\left(\sum_{j=1}^{m}X_{j}^{\prime }W_{j}T_{j}\right), \end{array}$$

where $W_{j}$ is a diagonal matrix containing the weights assigned to the effects of study *j*. In the classical application of the RVE inference method, weights ( $W_{j}$ ) can be approximated by either assuming that effect sizes are correlated because they have been calculated on the same sample (i.e., correlated effects) or assuming that effect sizes covary because they have been obtained under similar conditions: similar research groups, similar instruments and procedures, etc. (i.e., hierarchical effects). Formulas to obtain each of these types of weights can be found elsewhere.^20^ It should be noted that new approaches have been recently proposed where both types of dependency can be simultaneously modeled.^24^

After estimating the vector of moderator effects $\hat{\text{γ}}$ , their sampling variances (i.e., standard errors) are given by

(9) $$\begin{array}{r} V\left(\hat{\text{γ}}\right)={\left(\sum_{j=1}^{m}X_{j}^{\prime }W_{j}X_{j}\right)}^{-1}\left(\sum_{j=1}^{m}X_{j}^{\prime }W_{j}e_{j}e_{j}^{\prime }W_{j}X_{j}\right){\left(\sum_{j=1}^{m}X_{j}^{\prime }W_{j}X_{j}\right)}^{-1}, \end{array}$$

where $e_{j}$ is the observed vector of residuals of the *j*th study. In subsequent developments of this approach, Tipton^21^ and Pustejovsky and Tipton^23^ explored ways of further adjusting the estimated variances of the regression coefficients, $V\left(\hat{\text{γ}}\right),$ to keep the Type I error at the nominal level, especially when the number of studies synthesized is small (i.e., less than 40). In this regard, Tipton^21^ found a good performance for a combination of two small-sample corrections, namely the bias-reduced linearization adjustment proposed by Bell and McCaffrey^29^ applied on $V\left(\hat{\text{γ}}\right)$ , along with the Satterthwaite correction to the degrees of freedom of the *t*-distribution used to test the estimated regression coefficient.^30^ Specifically, Tipton^21^ proposed adding an adjustment matrix $A_{j}$ next to the cross-product of residuals:

(10) $$\begin{array}{r} V\left(\hat{\text{γ}}\right)={\left(\sum_{j=1}^{m}X_{j}^{\prime }W_{j}X_{j}\right)}^{-1}\left(\sum_{j=1}^{m}X_{j}^{\prime }W_{j}A_{j}e_{j}e_{j}^{\prime }A_{j}W_{j}X_{j}\right){\left(\sum_{j=1}^{m}X_{j}^{\prime }W_{j}X_{j}\right)}^{-1}. \end{array}$$

For a full description of the bias-reduced linearization adjustment (i.e., matrix $A_{j}$ ) and the Satterthwaite correlation to the degrees of freedom, we refer to the works of Tipton,^21^ Tipton and Pustejovsky,^32^ and Pustejovsky and Tipton.^23^ It should be noted that the Satterthwaite degrees of freedom are particularly sensitive to the unbalanced distribution of effect sizes across the categories of a categorical variable, especially in study-level covariates. Specifically, Tipton^21^ found that when the Satterthwaite degrees of freedom are smaller than 4, the Type I error rate can be inflated, making *p*-values unreliable.

Recently, Joshi et al.^26^ proposed combining the correlated-effects models with RVE method with bootstrapping techniques to enhance the power for detecting moderator effects while maintaining the Type I error rate at the nominal level. Among the various bootstrapping methods, Joshi et al.^26^ recommend using CWB,^33^ which accounts for the clustered structure of effect sizes within studies. This approach begins by fitting two models to the meta-analytic data: a null model, typically a standard random-effects model excluding moderator variables, and a full model that incorporates a moderator variable. A test statistic is then derived from the full model. Residual vectors from the null model are subsequently obtained, clustered by study. Then, for each bootstrap iteration, a random value (with mean 0 and variance 1) is generated for each study, and the corresponding residual vector is multiplied by this value. These modified residuals are added to the predicted values from the null model to create a new set of bootstrapped outcome scores. The full model is subsequently refitted using these scores, and a new test statistic is computed.

This process yields a *p*-value, calculated as the proportion of bootstrap test statistics that exceed the test statistic obtained from the original data analysis. This value is then used to determine whether the effect of the moderator variable is statistically different from zero. Technical details of this method, as well as relevant R code for its implementation, can be found in Joshi et al.^26^

## 3 Empirical example

To illustrate the properties of the methods discussed above, we use real meta-analytic data from the previously mentioned study by Yan et al.^2^ In this meta-analysis, the authors compared peer assessment with teacher assessment in enhancing academic performance. As previously presented, one potential moderator explored was whether students received feedback following their self-assessments. The results presented in Table 1 are based on a meta-regression in which the binary variable “feedback” (coded as 0 = no feedback; 1 = feedback) was included as a predictor of the standardized mean differences, which represent the relative efficacy of peer assessment compared to teacher assessment. It is important to note that this variable reflects a characteristic at the effect size level; that is, within a single study, some effect sizes were drawn from contexts where students received feedback on their peer assessments, while others came from contexts where no feedback was provided. Additionally, the distribution of effect sizes across the two categories was highly unbalanced: 259 effect sizes were drawn from scenarios where no feedback was provided to students, while only 78 effect sizes fell into the category where feedback was given.

The differences in *p*-values and estimates across methods are noteworthy. The multivariate approach indicates a statistically significant difference between categories, while the three-level model yields results approaching significance. In contrast, the three-level model with RVE and the version including an additional random effect both produce non-significant results. Moreover, the correlated-effects models with RVE and with RVE and CWB appear more conservative in this example. Overall, this brief illustration highlights how the choice of analytical method can influence meta-regression outcomes, with different approaches potentially leading to different conclusions. The data for this example and the R code used to generate the results in Table 1 are available at https://osf.io/czwtp/overview.

## 4 Method

### 4.1 Data generation

The R code to run the simulation studies and supplementary results are available at https://osf.io/czwtp/overview.

#### 4.1.1 Study-level moderator

Cohen’s *d* effect sizes were directly generated from a multivariate two-level model:

(11) $$\begin{array}{r} d_{ij}=\beta_{0j}+e_{ij} \end{array}$$

where $d_{ij}$ refers to the observed effect size *i* reported in study *j*, and $\beta_{0j}$ represents the overall population effect size of study *j*. The term $e_{ij}$ represents the random deviation of $d_{ij}$ from $\beta_{0j}$ due to the use of a sample of participants (e.g., random sampling error). The estimation procedure typically used in this model, namely ML or REML, assumes that the vector of residuals e within study *j* follows a multivariate normal distribution with mean 0 and with the following *I* × *I* variance–covariance matrix (**V**), with *I* being the total number of effect sizes within a study, so that

$$\begin{array}{r} \left[\begin{array}{c} e_{1j}\\ \begin{array}{c} e_{2j}\\ \begin{array}{c} \vdots \\ e_{Ij} \end{array} \end{array} \end{array}\right]∼MVN\left(\left[\begin{array}{c} 0\\ \begin{array}{c} 0\\ \begin{array}{c} \vdots \\ 0 \end{array} \end{array} \end{array}\right],\left[\begin{array}{ccc} {\sigma^{2}}_{e_{1}} & & \;\\ \sigma_{e_{1}e_{2}} & {\sigma^{2}}_{e_{2}} & \\ \begin{array}{c} \vdots \\ \sigma_{e_{1}e_{I}} \end{array} & \begin{array}{c} \vdots \\ \sigma_{e_{2}e_{I}} \end{array} & \begin{array}{c} \begin{array}{cc} \ddots & \end{array}\\ \begin{array}{cc} \ldots & {\sigma^{2}}_{e_{I}} \end{array} \end{array} \end{array}\right]\right). \end{array}$$

In the main diagonal of the variance–covariance matrix, we find the sampling variance of each effect size, which was calculated following the formula provided by Gleser and Olkin^34^:

(12) $$\begin{array}{r} \sigma_{e_{ij}}^{2}=\frac{n_{1}+n_{2}}{n_{1}n_{2}}+\frac{\delta_{ij}^{2}}{2\left(n_{1}+n_{2}\right)}, \end{array}$$

where $n_{1}$ and $n_{2}$ represent the sample sizes of the experimental and control groups, and $\delta_{ij}$ refers to the population effect of effect size *i* within study *j*. Moreover, covariances between effect sizes (e.g., $\sigma_{e_{1}e_{2}}$ ) are specified outside the main diagonal. For this study, we have assumed that effect sizes reported in the same study referred to different types of outcomes. Therefore, the covariances were computed using the formulas established by Gleser and Olkin^34^ for Cohen’s *d* effect sizes for this scenario:

(13) $$\begin{array}{r} \sigma_{e_{ij}e_{ij\prime }}=\frac{n_{1}+n_{2}}{n_{1}n_{2}}\rho_{ijij\prime }+\frac{\delta_{ij}\delta_{ij\prime }\rho_{ij\;ij\prime }^{2}}{2\;\left(n_{1}+n_{2}\right)} \end{array}$$

where $\delta_{ij}$ and $\delta_{ij\prime }$ refer to the population effects of two different effect sizes reported within study *j*, and $\rho_{ij\;ij\prime }$ refers to the correlation between outcome variables.

At Level 2, the study-specific population effects $\beta_{0j}$ is a function of a study-dummy (moderator) variable $X_{1j}$ :

(14) $$\begin{array}{r} \beta_{0j}=\gamma_{00}+\gamma_{01}X_{1j}+u_{0j}, \end{array}$$

where $\gamma_{00}$ represents the overall effect size for category 0 of the moderator variable $X_{1j}$ , $\gamma_{01}$ is the difference between the overall effects from both categories, and $\gamma_{00}+\gamma_{01}$ is the overall effect size for category $X_{1j}$ = 1. The study-specific random effects, $u_{0j}$ , represent the random deviation of $\beta_{0j}$ from the overall effect size $\gamma_{00}$ due to between-studies variation. The random effect $u_{0j}$ is normally distributed with mean zero and variance $\sigma_{u_{0}}^{2}$ , which stands for the (residual) between-studies variance around the effect of $\gamma_{00}$ once the effect of $X_{1j}$ has been taken into account. Note that in this model, it is assumed that outcomes of the same type share a common population effect size.

Cohen’s *d* was not corrected to Hedges’ *g*, as Lin and Aloe^35^ found that using Cohen’s *d* as an estimator of population effect delta generally leads to less bias in the meta-analytic results than Hedges’ *g*. Also, following the recommendations of Lin and Aloe,^35^ the formula to calculate the sampling variance of Cohen’s *d* was the one in Equation (3) but replacing the population term $\delta_{ij}^{2}$ with $d_{ij}^{2}$ . Finally, it should be noted that by generating Cohen’s *d* directly, the results of this simulation can be generalized to effect sizes that are approximately normally distributed (e.g., Fisher’s *Z* or log-transformed odds ratio).

#### 4.1.2 Effect size-level moderator

Cohen’s *d* effect sizes were directly generated from a multivariate two-level model:

(15) $$\begin{array}{r} d_{ij}=\beta_{0j}X_{0ij}+\beta_{1j}X_{1ij}+e_{ij}, \end{array}$$

where $X_{0ij}$ and $X_{1ij}$ are dummy-coded variables indicating the category of the moderator variable to which the effect size pertains (coded as 1), and $e_{ij}$ is a vector of residuals with *I × I* variance–covariance matrix. At the second level:

(16) $$\begin{array}{r} \left\{ \begin{array}{l} \beta_{0j}=\gamma_{00}+u_{0j}\\ \beta_{1j}=\gamma_{10}+u_{1j}, \end{array}\right. \end{array}$$

where $\gamma_{00}$ and $\gamma_{10}$ represent, respectively, the overall effect for category 0 and the overall effect for category 1. The study-specific random effects, $u_{0j}$ and $u_{1j}$ , are assumed to follow a bivariate normal distribution:

$$\begin{array}{r} \left[\begin{array}{c} u_{0j}\\ u_{1j} \end{array}\right]∼MVN\;\left(\left[\begin{array}{c} 0\\ 0 \end{array}\right],\left[\begin{array}{cc} \sigma_{u_{0}}^{2} & \\ \sigma_{u_{0}u_{1}} & \sigma_{u_{1}}^{2} \end{array}\right]\right), \end{array}$$

where the variances $\sigma_{u_{0}}^{2}$ and $\sigma_{u_{1}}^{2}$ stand for the between-studies variances of the population effect sizes $\gamma_{00}$ and of the differences between effects, $\gamma_{10}$ , respectively. These between-studies variances were always equal, meaning that the assumption of homoscedasticity was satisfied. Additionally, it is important to note that this model operates under the assumption that differences between categories may vary across studies. This implies that while one study may show no differences between categories, another may indeed reveal variations. We adopt this approach because there is no compelling reason to assume that overall effects within categories of a moderator remain consistent across studies. Similar to how a random-effects model allows the overall effect to randomly vary across studies, the effects within a moderator category can also exhibit variability across studies. In cases where such variation occurs, we must acknowledge that the differences between effects, denoted as $\gamma_{10}$ , can also vary across studies.

The correlation selected to calculate the covariances for between-study residuals ( $\sigma_{u_{0}u_{1}}$ ) was set to 0.4, which is an intermediate value.^22^ ^,^ ^28^ This correlation was not varied in the present simulation because these two previous studies have found no effect of this factor on simulation results.

The difference in overall effects across categories is represented by $\gamma_{01}$ when the moderator effect is at the study level, and by $\left(\gamma_{10}-\gamma_{00}\right)$ when it operates at the effect size level. To avoid confusion, for the remainder of the article, we will refer to the overall effect of the first category as $\gamma_{0}$ , and to the moderator effect of the second category as $\gamma_{1}$ , regardless of whether it pertains to a study-level or effect size-level characteristic.

### 4.2 Simulation factors

#### 4.2.1 Number of studies (k)

In the review of multilevel meta-analyses carried out by Fernández-Castilla et al.,^36^ the Q1, Q2, and Q3 number of studies in multilevel meta-analysis of different fields were around 18, 35, and 53. Previous simulation studies have found that 40 studies are normally enough to get unbiased estimates.^9^ ^,^ ^22^ Therefore, also following the procedure of Tipton^21^ and López-López et al.,^9^ we generated meta-analyses of 20 and 40 studies, corresponding to (approximately) Q1 and Q2.

#### 4.2.2 Number of effect sizes within studies (m)

According to Fernández-Castilla et al.^36^ primary studies reporting multiple results rarely include the same number of outcomes, and, on average, they include around 3 effect sizes. Tipton et al.^25^ found that the average number of effect sizes per study was around 4.5 in meta-analysis in the psychology and education areas. These unbalanced distributions of effect sizes across studies were taken into account in the simulation by generating different number of effect sizes across studies. First, the average number of effect sizes per study was set to be 3 or 5 ( $\overset{―}{m}$ = {3, 5}). Then, a number was extracted from a Poisson distribution as

$$\begin{array}{r} m_{j}∼\min\left\{1+Poisson\left(\overset{―}{m}-1\right),\left(\overset{―}{m}*2+1\right)\right\}. \end{array}$$

This approach is similar to the one Joshi et al.^26^ used to generate their data. When $\overset{―}{m}=3$ , the number of effect sizes per study ranged between 1 and 6, and when $\overset{―}{m}=5$ , the number of effect sizes per study ranged between 1 and 9.

#### *Correlation between effect sizes (* ρ *)*

4.2.3

Effect sizes from the same study could be moderately ( ρ=0 .4) or largely correlated ( ρ=0 .8). These values are consistent with those selected in previous simulation studies. For instance, López-López et al.^9^ chose an average correlation of 0.3 (moderate) or 0.7 (large), whereas Joshi et al.^26^ and Tipton^21^ chose correlations of 0.5 and 0.8 for moderate and large correlations, respectively. Following the procedure of Joshi et al.,^26^ correlations were varied across studies by extracting a correlation value from a beta distribution as follows:

$$\begin{array}{r} r_{j}∼Beta\left(\rho \upsilon ,\left(1-\rho \right)\upsilon \right), \end{array}$$

where υ was always set to 50 to generate moderate heterogeneity across correlations.

#### 4.2.4 Sample size (n)

In the review of multilevel meta-analyses carried out by Fernández-Castilla et al.,^36^ the median sample size (*n*) in primary studies was around 100, with the first quartile being around 35.^4^ As sample sizes tend to vary significantly among primary studies, we generated random numbers from a lognormal distribution, either LnN(3.91, 0.7) or LnN(4.61, 0.7), reproducing the sample sizes observed in the review. When the mean of the lognormal distribution was 3.91, the average total sample size was 45, and this value ranged from approximately 15 to 200. When the mean of the lognormal distribution was 4.61, the average total sample size was 100, and this value approximately ranged from 52 to 500.

#### 4.2.5 Type of moderator variable

The binary moderator variable could explain characteristics of the studies (study-level moderator) or characteristics of the effect sizes within study (effect size-level moderator).

#### 4.2.6 Overall effect of each category

The overall effect for the reference category ( $\gamma_{0}$ ) was always 0, and the difference between categories, $\gamma_{1}$ , could be 0 as well (this condition serves to study Type I error), or 0.10, 0.30, and 0.50 (these conditions serve to study power). These values are similar to those selected in previous simulations; for instance, Rubio-Aparicio et al.^10^ generated differences of 0.2, 0.4, and 0.6, whereas Joshi et al.^26^ generated differences of 0.1, 0.3, and 0.5. Also, these values represent a realistic range of Cohen’s *d* indices typically observed in meta-analyses from different fields.^36^ Additionally, as described by Cafri et al.^5^ according to a review of 81 meta-analysis, the median difference between overall effects of the two categories of a moderator is 0.16 (*r* = 0.08).

#### 4.2.7 Distribution of effects across the categories of the dummy moderator variable

A key focus of this study was to investigate how an uneven distribution of effect sizes across the categories of the moderator variable impacts parameter estimates, the Type I error rate, and statistical power. Three scenarios were examined: balanced (50%–50%), unbalanced (30%–70%), and very unbalanced (10%–90%) distributions of effect sizes across categories of the binary moderator variable. When the moderator variable was at the study level, entire studies were assigned to one category or the other based on the specified percentages. In contrast, when the moderator was at the effect size level, individual effect sizes within studies were randomly assigned to each category according to the same proportions.

#### *(Residual) between-studies variance (* ${\hat{\sigma }}_{u_{res}}^{2}$ *)*

4.2.8

To simplify the analysis, we selected two values for between-studies variance representing moderate and high variability: 0.10 for moderate and 0.30 for high variability. These values align with those reported by Fernández-Castilla et al.^36^ in multilevel meta-analyses across various fields and match conditions used in previous simulations.^9^ ^,^ ^26^ When the moderator variable was at the effect size level, the overall effects for each category could vary across studies ( $\sigma_{u_{0j}}^{2}$ and $\sigma_{u_{1j}}^{2}$ ), with both variances set to 0.05 or 0.15 under an assumption of homoskedasticity. The correlation between random study effects was fixed at 0.4, based on prior simulations^22^ ^,^ ^28^ showing no significant impact of this correlation. We chose this intermediate value as representative of earlier studies. Please note that in the scenario where the moderator variable referred to an effect size-level characteristic, the total variance became slightly smaller, 0.24 and 0.08, respectively, due to the 0.4 correlation between study effects.

All these settings resulted in a total of 768 scenarios, with 1,000 replications conducted for each of them.

### 4.3 Data analysis

In this section, we outline the statistical techniques that were applied to run the meta-regression models.

#### 4.3.1 Multivariate method

Although this method is not central to the present study, primarily due to the frequent unavailability of the information needed to calculate the covariance among effect sizes within studies, we still find it valuable to explore its performance, as this approach has been increasingly implemented following the approximation of a variance–covariance matrix.^37^ When the moderator variable referred to study-level characteristics, the multivariate model implemented was the one specified in Equations (11) and (14), consistent with the data generation process. In this case, the variance–covariance matrix of the observed effect sizes was approximated using the same correlation value specified during data generation (either 0.4 or 0.8, depending on the simulation condition), with the data function impute_covariance_matrix() from the *clubSandwich* package.^38^ When the moderator variable referred to effect size-level characteristics, we implemented a multivariate model similar to that specified in Equations (15) and (16), with a simplification applied to Equation 16. Specifically, we removed the term $u_{1j}$ , implying that the difference between moderator categories was assumed to be constant across studies. In this scenario, the approximate variance–covariance matrix was constructed using the average correlation between effect sizes within studies, which varied depending on whether the effect sizes belonged to the same moderator category. This modified multivariate model was used because, in practice, it is uncommon to specify a model that includes a random effect for the difference between moderator categories. It is also important to note that Assink and Wibbelink^37^ proposed a more complex multivariate model with an additional (third) level. The significance of the moderator effect ( ${\text{γ}}_{1}$ ) was evaluated using a Wald test.

#### 4.3.2 Three-level models

When the moderator variable reflected study-level characteristics, we fitted the three-level model specified in Equations (1)–(3). For moderator variables that represented effect size-level characteristics, two versions of the three-level models were applied. The first corresponds to the model defined in Equations (1), (4), and (5), where the moderator effect is assumed to be constant across studies (a specification commonly used in published meta-analyses). The second model, defined in Equations (1), (4), and (6), allows the moderator effect to vary across studies, which more accurately reflects the data generation process.

In all these models, the statistical significance of the moderator effect ${\text{γ}}_{1}$ was evaluated using Wald tests (based on Z statistics) with a nominal level of 0.05. LRTs were only used for the traditional three-level model (using Equations (1)–(3) for study-level moderator, and Equations (1), (4), and (5) for effect size-level moderator) by comparing the deviance of the null model with that of the model including the moderator.

However, for the extended three-level model including an additional study-level random effect for the moderator, the significance of the moderator effect was assessed using only Wald tests. This is because comparing the null model to a model that includes both the moderator and its study-level random effect involves estimating three additional parameters: the fixed moderator effect, its variance, and the covariance between study-level random effects. As a result, a significant LRT result could reflect the influence of any of these components, making it difficult to isolate the specific contribution of the moderator effect. The *metafor* package^39^ was used for these analyses.

Last, within this framework, we also implemented the combination of three-level models and RVE. To do this, we first fit the three-level model specified in Equations (1)–(3) (for study-level characteristics) or the model in Equations (1), (4), and (5) (for effect size-level characteristics). RVE inferential method was then applied to adjust the variance–covariance matrix obtained from the three-level model using a sandwich estimator. Inference in this case was based on *t-*tests with Satterthwaite-corrected degrees of freedom. These analyses were conducted using the metafor^39^ and clubSandwich^38^ R packages.

#### 4.3.3 Correlated-effects model with RVE inferential method

This method was implemented using correlated effects, and small-sample corrections were applied as recommended by Tipton^21^ and Pustejovsky and Tipton,^23^ specifically the bias-reduced linearization adjustment and the Satterthwaite degrees of freedom correction. The *t*-statistic, along with the corrected degrees of freedom, was used to assess the significance of the regression coefficients ( α=0.05) . These analyses were conducted using the *robumeta* package.^40^

Finally, within this framework, we implemented this method followed by the application of CWB. This approach was applied both with and without the bias-reduced linearization adjustment. The method produces an F-statistic to evaluate the pre-specified hypothesis. All analyses were conducted using the *wildmeta* package R.^41^

### 4.4 Analysis of the simulation results

To identify the simulation conditions associated with different results (e.g., bias, mean squared error, Type I error, and power), we conducted analyses of variance (ANOVAs), where the independent variables were the simulation factors. We considered simulation factors with an eta squared ( $\eta^{2}$ ) value exceeding 0.14 as influential.^42^

#### 4.4.1 Fixed effects

To assess the accuracy of the overall effects for each category of the binary variable $(\gamma_{0}$ and $\gamma_{1}$ ), we calculated the bias (i.e., the observed estimate minus true value). Relative bias is not used here because the additive nature of the parameter makes absolute bias a more meaningful measure (the relative bias would be overly sensitive to shifts in scale) and because it cannot be calculated when the overall effect of the first category, $\gamma_{0}$ , is set to zero. Additionally, the mean squared error (MSE) was calculated for the overall effects of each category of the categorical variable.

#### 4.4.2 Type I error

The Type I error was examined under the condition where the difference between the overall effects ( $\gamma_{1}$ ) was zero. Specifically, we calculated the proportion of iterations in which a significant difference between effects was detected, despite the true difference being zero. Due to simulation error, it is likely that the Type I error is not exactly 0.05 for each combination of conditions. To assess whether the Type I error falls within reasonable bounds given the simulation error, we constructed confidence intervals around the nominal value of α using the following formula:

$$\begin{array}{r} CI=0.05\mp 1.96\;∙\;\sqrt{\frac{0.05\left(1-0.05\right)}{1000}\;}. \end{array}$$

This resulted in a confidence interval ranging from 0.036 (lower bound) to 0.064 (upper bound).

#### 4.4.3 Power

Statistical power was evaluated in those conditions where the difference between the overall effects of the moderator categories ( $\gamma_{1}$ ) was different from zero. Power was calculated as the percentage of iterations in which the *p*-value for the difference between effects was below the nominal level of 0.05.

## 5 Results

Across all results, the correlated-effects models with RVE and CWB, both with and without the bias-reduced linearization adjustment, produced identical outcomes. Therefore, we report results for these methods without adjustment.

To describe results for non-convergence rates, fixed-effects bias, and MSE, we present only the results for the multivariate method, the three-level model, and the correlated-effects models with RVE, since the remaining methods (three-level models with RVE and correlated-effects models with RVE and CWB) affect only the inference provided by these methods, not the fixed-effect estimates.

### 5.1 Study-level moderator

#### 5.1.1 Non-convergence

The three-level model failed to converge in 62 out of 384,000 iterations, whereas the multivariate method failed to converge in only 2 out of 384,000 iterations. In contrast, correlated-effects models with RVE method systematically converged across all conditions and iterations. Non-convergence in the three-level model was most frequent when sample size was large (85.48% of the non-convergence iterations), between-study variance was high (74.19%), the number of studies was small (69.34%), and within-study correlation was low ( ρ = 0.4, 66%).

#### 5.1.2 Bias of the fixed effects

The mean bias of $\gamma_{0}$ ranged from −0.032 to 0.021, and the mean bias of $\gamma_{1}$ ranged from −0.027 and 0.013, indicating that both parameters were generally underestimated rather than overestimated under certain conditions. When running an ANOVA with the bias of $\gamma_{0}$ , no condition exhibited an *η^2^* greater than 0.14. Nevertheless, the condition with the higher (though still low) *η^2^* was the distribution of effect sizes, with a higher biased observed in $\gamma_{0}$ when effect sizes were very unbalanced distributed (mean bias of $\gamma_{0}$ in this condition was 0.001). For the parameter $\gamma_{1}$ , the true value of $\gamma_{1}$ was the factor that most strongly affected its bias. The greater the value of $\gamma_{1}$ , the greater the underestimation of $\gamma_{1}$ (the mean bias when $\gamma_{1}$ = 0.5 was −0.008).

Figures representing the bias of $\gamma_{0}$ and $\gamma_{1}$ for each method can be found in the Supplementary Material (Figures S1 and S2, respectively), alongside a spreadsheet with the bias for each combination of conditions (*Study-level_bias and MSE.xlsx*).

#### 5.1.3 Mean squared error

To better understand these results, it is important to note that under unbalanced conditions, the category with fewer effect sizes was associated with $\gamma_{0}$ . Consequently, under these conditions, fewer effect sizes were available for estimating $\gamma_{0}$ , which explains why MSE was systematically larger for this parameter.

Both the three-level model and correlated-effects models with RVE led to similar average MSEs for both $\gamma_{0}$ (MSE = 0.046) and $\gamma_{1}$ (MSE = 0.014), while the multivariate method led to a slightly smaller MSE (MSE for $\gamma_{0}$ = 0.045 and for $\gamma_{1}$ = 0.013). Overall, the ANOVA analyses showed that for $\gamma_{0}$ and $\gamma_{1},$ the MSE was lower when the variance between studies was small (η^2^ = 0.18 for $\gamma_{0}$ and η^2^ = 0.43 for $\gamma_{1}$ ) and the number of studies was large (η^2^ = 0.12 for $\gamma_{0}$ , η^2^ = 0.30 for $\gamma_{1}$ ; see Table S1 in the Supplementary Material). For all methods, the MSE of $\gamma_{0}$ was additionally affected by the uneven distribution of effect sizes across subgroups (η^2^ = 0.45), whereas this factor did not seem to affect the MSE of $\gamma_{1}$ (see Table S1 in the Supplementary Material). In the Supplementary Material, we provide the MSE for each method and combination of conditions (*Study-level_bias and MSE.xlsx*).

#### 5.1.4 Type I error

Table 2 provides the average Type I error rate observed for each method in each category of each simulation condition.

**Table 2:** Mean Type I error rate disaggregated by method and simulation conditions Table 2 Long description.

Study-level moderator						
	Multivariate	Three-level model		RVE-based		
	Wald test	Wald test	LRT	Three-level with RVE	CE – RVE	CE – CWB
Mean	0.062	0.062	0.062	0.040	0.040	0.044
Number of studies						
*k* = 20	**0.067**	**0.066**	**0.067**	**0.033**	**0.033**	**0.035**
*k* = 40	0.058	0.058	0.058	0.047	0.046	0.053
Sample size						
Mean *n* ≈ 45	**0.065**	0.063	0.064	0.042	0.041	0.046
Mean *n* ≈ 100	0.060	0.060	0.061	0.038	0.037	0.042
Number of outcomes						
Around 3	0.062	0.061	0.062	0.039	0.039	0.043
Around 5	0.063	0.062	0.063	0.041	0.040	0.044
Distribution across categories					
Balanced	0.061	0.060	0.061	0.050	0.050	0.049
Unbalanced	0.063	0.062	0.062	0.049	0.049	0.053
Very unbalanced	0.064	0.063	0.063	**0.021**	**0.021**	**0.031**
Correlation between ESs						
ρ= 0.4	0.063	0.063	0.063	0.041	0.040	0.045
ρ= 0.8	0.061	0.061	0.061	0.039	0.039	0.043
Total variance						
Small – 0.10	0.062	0.061	0.061	0.039	0.039	0.043
Large – 0.30	0.062	0.063	0.063	0.041	0.040	0.045

*Note:* LRT = likelihood ratio test; CE = correlated-effects models; RVE = robust variance estimation; CWB = cluster wild bootstrapping. Values in bold indicate Type I errors outside the confidence interval that accounts for simulation error.

As shown in Table 2, the multivariate method and three-level models (when not combined with RVE) led to slightly inflated Type I error rates, although these rates were mostly within the confidence interval for simulation error. The application of three-level models with RVE, the correlated-effects models with RVE, and correlated-effects models with RVE and CWB resulted in Type I error rates below the nominal level. An ANOVA conducted to determine which simulation conditions were associated with the Type I error indicated that, in addition to the type of method applied (η^2^ = 0.25), the distribution of effect sizes across categories also affected the Type I error rates (η^2^ = 0.10).

**Figure 1:**
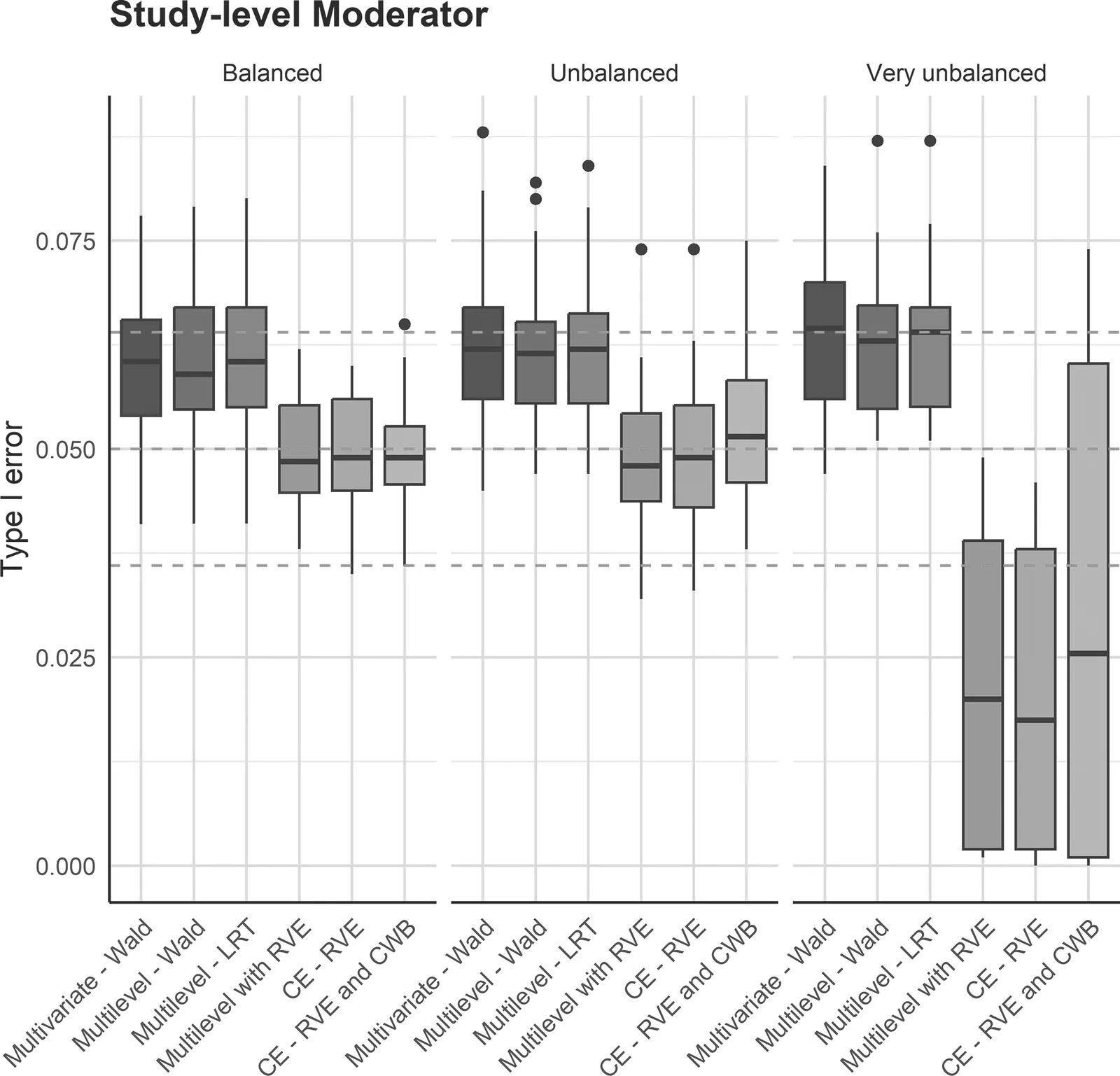
Type I error distribution across methods, disaggregated by the degree of imbalance in the distribution of effect sizes across the moderator categories. Figure 1 Long description.

Figure 1 shows the distribution of Type I error rates by method for different distributions of effect sizes. The multivariate method and three-level models (with both Wald and LRT tests) led to slightly inflated Type I errors under some conditions, especially if effect sizes were very unbalanced distributed across the categories of the moderator variable, whereas the RVE-based methods showed good control of Type I error across most conditions. However, these methods became too conservative when effect sizes were very unevenly distributed across the categories of the moderator (90%–10%), especially three-level models with RVE and the correlated-effects model with RVE.

As the next step, we focused specifically on the Type I error rates observed when applying a multivariate method or a three-level model (using both Wald and LRT tests). The number of studies included in the meta-analysis emerged as the most influential simulation factor for both methods. In multilevel models, the Type I error rate increased to 0.087 when *k =* 20, but remained within the expected range (0.036–0.064) when the number of studies was increased to 40. In the multivariate method, some inflation in the Type I error rate (up to 0.070) was observed, even with 40 studies, particularly when the sample size was small (*n* = 45). The Type I error rate disaggregated by each method and combination of conditions can be found in the Supplementary Material (*Study-level_Type I error.xlsx*).

#### 5.1.5 Power

Table 3 provides the average power observed for each method in each category of each simulation condition. Some expected patterns are observed, such as an increase in power with a higher number of studies, a greater difference between categories, a decrease in total variability, and a more balanced distribution of effect sizes across the categories of the moderator. The rest of the simulation factors did not seem to substantially affect the power.

**Table 3:** Power disaggregated by the simulated conditions and method Table 3 Long description.

	Study-level moderator					
	Multivariate	Three-level model		RVE-based		
	Wald test	Wald test	LRT	Three-level with RVE	CE – RVE	CE – CWB
Mean		0.360	0.361	0.290	0.290	0.300
Number of studies						
*k* = 20	0.296	0.295	0.297	0.204	0.206	0.204
*k* = 40	0.427	0.424	0.426	0.375	0.374	0.395
Sample size						
Mean *n* ≈ 45	0.348	0.347	0.349	0.278	0.278	0.287
Mean *n* ≈ 100	0.374	0.372	0.373	0.301	0.302	0.311
Number of outcomes						
Around 3	0.359	0.358	0.359	0.288	0.289	0.297
Around 5	0.364	0.362	0.363	0.291	0.291	0.301
Difference between categories						
$\gamma_{10}=$ 0.10	0.096	0.096	0.095	0.067	0.067	0.072
$\gamma_{10}=$ 0.30	0.348	0.349	0.347	0.276	0.276	0.286
$\gamma_{10}=$ 0.50	0.640	0.640	0.638	0.526	0.526	0.540
Distribution across categories						
Balanced	0.438	0.437	0.439	0.412	0.413	0.412
Unbalanced	0.404	0.402	0.404	0.360	0.361	0.360
Very unbalanced	0.242	0.240	0.242	0.097	0.096	0.126
Correlation between ESs						
0.4	0.369	0.368	0.369	0.297	0.297	0.206
0.8	0.354	0.352	0.353	0.283	0.283	0.293
Total variance						
Small – 0.10	0.451	0.449	0.450	0.366	0.366	0.377
Large – 0.30	0.272	0.271	0.273	0.214	0.214	0.222

*Note:* LRT = likelihood ratio test; CE = correlated-effects models; RVE = robust variance estimation; CWB = cluster wild bootstrapping.

In general, the multivariate method and three-level models (using both Wald and LRT tests) exhibited slightly higher power rates. Interestingly, when RVE was applied in combination with three-level models, the power rates were only marginally affected, except in cases where effect sizes were very unevenly distributed across the categories of the moderator variable. This finding is important because it demonstrates that the combination of three-level models and RVE adequately controls Type I error while only minimally affecting power.

The most noticeable difference in power across methods occurs under the “distribution across categories” conditions. On average, when effect sizes were very unevenly distributed, the power rate of RVE-based methods was nearly one-third of that observed with multilevel approaches and with the multivariate method. Overall, the highest power observed for all methods (approximately 0.60) occurred when the difference between the overall effects across categories was large (0.5).

**Figure 2:**
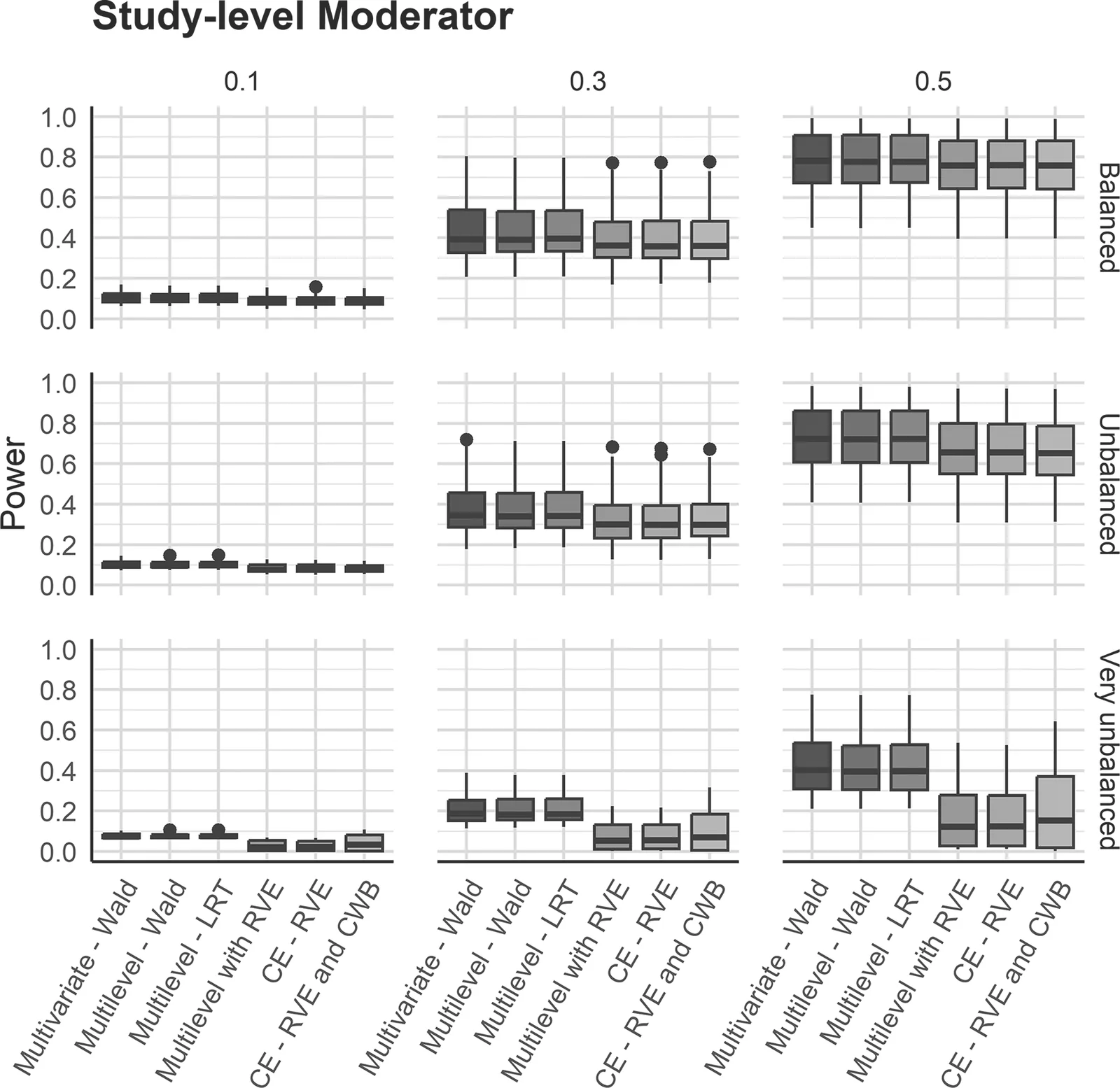
Statistical power across methods, disaggregated by the magnitude of differences between categories and the degree of imbalance in effect size distributions across moderator categories. Figure 2 Long description.

An ANOVA was conducted entering power rates as dependent variable. The simulation factors exerting a stronger effect on power were the difference between categories (η^2^ = 0.49) and the distribution of effect sizes across categories (η^2^ = 0.14). The distribution of power rates across these conditions is shown in Figure 2. The average power disaggregated by each method and combination of simulation factors can be found in the Supplementary Material (*Study-level_Power.xlsx*).

As shown in Figure 2, when the distribution of effect sizes was balanced across categories and the difference between categories is large ( $\gamma_{1}$ = 0.5), all methods showed the highest power rates. However, as the distribution of effect sizes became more uneven, power decreased, particularly for the RVE-based methods, even when the difference between categories was substantial. Under these conditions (highly unbalanced effect size distribution across categories and a large difference between categories), multilevel approaches demonstrate higher power but remain insufficient. The lowest power across all methods was observed when the difference between categories was small, regardless of how effect sizes were distributed across categories.

Other interesting results are that correlated-effects models with RVE and CWB only slightly improved power compared to traditional RVE, and that when using three-level models, the Wald and LRT tests showed similar power properties.

### 5.2 Effect size-level moderator

#### 5.2.1 Non-convergence

Under this scenario, the three-level model did not converge in 161 out of the 384,000 iterations (0.04%), whereas the three-level model that allowed the moderator effect to vary across studies did not converge in 427 out of the 384,000 iterations (0.11%). The multivariate model experienced only a few convergence issues, with 86 non-converging iterations (0.02% of their respective iterations). The correlated-effects models with RVE method always converged.

A separate analysis of dataset characteristics associated with non-convergence rates revealed that, for both the multivariate method and multilevel models (with or without a random effect for the moderator effect), non-convergence was more likely when the number of studies was small (*k* = 20), effect sizes were highly unbalanced across moderator categories, and the average number of outcomes per study was low (*m* = 3).

#### 5.2.2 Bias of the fixed effects

The mean bias of $\gamma_{0}$ ranged from −0.024 to 0.012, and the mean bias of $\gamma_{1}$ ranged from −0.035 and 0.008, indicating that, as with study-level moderators, both parameters were generally underestimated rather than overestimated. The negative bias for $\gamma_{1}$ was more pronounced than when the moderator variable referred to a study-level characteristic.

ANOVAs performed on the bias of $\gamma_{0}$ and $\gamma_{1}$ showed that none of the conditions predicted these biases. Still, the factor that most strongly influenced the bias of $\gamma_{0}$ was the type of method used: the largest mean bias for $\gamma_{0}$ was observed when the multivariate model was implemented (bias = −0.003). In contrast, the factor with the greatest influence on $\gamma_{1}$ was the true value of $\gamma_{1}$ : as the true value of $\gamma_{1}$ increased, the bias also became larger in magnitude (mean bias when $\gamma_{1}=$ 0.5 is −0.007).

Graphs illustrating the bias of $\gamma_{0}$ and $\gamma_{1}$ for each method are provided in the Supplementary Material (Figures S3 and S4, respectively), along with a spreadsheet detailing the bias and RB for every combination of conditions (*Effect size-level_bias and MSE.xlsx*).

#### 5.2.3 Mean squared error

The method that led to the highest efficiency when the moderator variable referred to a within-study characteristic was the three-level model allowing the moderator effect to vary across studies (MSE for $\gamma_{0}$ = 0.012 and for $\gamma_{1}$ = 0.006). All the other methods led to similar MSEs (MSE for $\gamma_{0}$ = 0.013 and for $\gamma_{1}$ = 0.006). Results from an ANOVA showed that, similar to the results obtained for study-level moderators, the MSE peaked when the between-studies variance was low ( $\eta^{2}$ = 0.17 for $\gamma_{0}$ and $\eta^{2}$ = 0.45 for $\gamma_{1})$ , and when the number of studies was large ( $\eta^{2}$ = 0.16 for $\gamma_{0}$ and $\eta^{2}$ = 0.39 for $\gamma_{1}$ , see Table S1 in the Supplementary Material), and the MSE of $\gamma_{0}$ increased in conditions where effect sizes were unbalanced distributed across the categories of the moderator ( $\eta^{2}$ = 0.34), whereas this factor did not affect MSE of $\gamma_{1}.$

#### 5.2.4 Type I error

Table 4 provides the average Type I error for each method and condition.

**Table 4:** Average Type I error rates disaggregated by method and simulated conditions Table 4 Long description.

	Effect size-level moderator						
	Multivariate	Three-level model			RVE-based		
	Wald	Wald	LRT	Wald (random)	Three-level with RVE	CE – RVE	CE – CWB
Mean	**0.493**	**0.271**	**0.265**	**0.084**	0.059	0.047	0.049
Number of studies							
*k* = 20	**0.487**	**0.267**	**0.259**	**0.093**	0.057	0.045	0.048
*k* = 40	**0.500**	**0.275**	**0.270**	**0.075**	0.061	0.049	0.049
Sample size							
Mean *n* ≈ 45	**0.440**	**0.236**	**0.230**	**0.080**	0.058	0.048	0.049
Mean *n* ≈ 100	**0.546**	**0.307**	**0.299**	**0.087**	0.060	0.047	0.048
Number of outcomes							
Around 3	**0.458**	**0.231**	**0.223**	**0.090**	0.059	0.048	0.049
Around 5	**0.528**	**0.312**	**0.307**	**0.078**	0.059	0.046	0.049
Distribution across categories							
Balanced	**0.522**	**0.273**	**0.267**	**0.072**	0.062	0.049	0.050
Unbalanced	**0.502**	**0.270**	**0.263**	**0.073**	0.059	0.048	0.050
Very unbalanced	**0.455**	**0.270**	**0.266**	**0.107**	0.056	0.044	0.047
Correlation bet. within ESs							
0.2–0.4	**0.419**	**0.267**	**0.259**	**0.085**	0.059	0.048	0.050
0.6–0.8	**0.567**	**0.276**	**0.270**	**0.083**	0.059	0.046	0.048
Total variance							
Small – 0.10	**0.407**	**0.211**	**0.206**	**0.079**	0.059	0.047	0.049
Large – 0.30	**0.579**	**0.332**	**0.324**	**0.088**	0.059	0.047	0.048

*Note:* LRT = likelihood ratio test; CE = correlated-effects models; RVE = robust variance estimation; CWB = cluster wild bootstrapping. Values in bold indicate Type I errors outside the confidence interval that accounts for simulation error.

As shown, employing a three-level or a multivariate model assuming a common moderator effect across studies resulted in severely inflated Type I errors. This pattern was observed across all combinations of conditions when the categorical moderator described an effect size-level characteristic. The three-level model allowing the moderator effect to vary across studies led to much lower Type I error rates, though it remained overly liberal, especially under certain conditions.

To examine the factors influencing Type I error when the three-level model allowed the moderator effect to vary across studies, we isolated these results and conducted an ANOVA, using Type I error rates as the dependent variable and the simulation factors as independent variables. The distribution of effect sizes across the categories of the moderator variable ( $\eta^{2}=$ 0.33) and the number of studies ( $\eta^{2}=$ 0.13) were the most influential factors. When the number of studies was large (*k* = 40) and distribution of effects was even (50%–50%), the average Type I error was close to the nominal level (0.064). However, when the number of studies was small (*k* = 20) and the distribution of effects was very uneven (10%–90%), the Type I error rate was especially inflated (0.119).

Another important result is that three-level models with RVE led to average Type I errors that were practically at nominal level. The correlated-effects models with RVE, whether the traditional form or with CWB, resulted in Type I errors close to 0.05 and within the established confidence intervals across all 384 combinations of conditions. This indicates that implementing either the traditional RVE method or the CWB variant ensures adequate control of Type I error.

Table 4 shows that no simulation factor seemed to affect the Type I error of RVE-based approaches. However, the amount of variability among effect sizes did affect the Type I error of multilevel-based approaches: the larger the variability, the higher the Type I error. Other factors, including the distribution of effect sizes across the moderator categories, did not seem to influence the Type I error when the moderator variable referred to an effect size-level variable (see Figure S5 in the Supplementary Material). The Type I error rate disaggregated by each method and combination of conditions can be found in the Supplementary Material (*Effect size-level_Type I error.xlsx*).

#### 5.2.5 Power

The mean power, segmented by method and simulation condition, is presented in Table 5. In this analysis, we excluded the power results from the three-level model assuming a constant moderator effect across studies, as well as from the multivariate method, due to the inflated Type I error rates observed in these models.

**Table 5:** Mean power segmented by method and simulation condition Table 5 Long description.

	Effect size-level moderator			
	Three-level model (random)	RVE-based methods		
	Wald	Three-level	CE –	CE –
		with RVE	RVE	CWB
Mean	0.680	0.592	0.570	0.574
Number of studies				
*k* = 20	0.628	0.517	0.492	0.497
*k* = 40	0.731	0.667	0.648	0.651
Sample size				
Mean *n* ≈ 45	0.662	0.578	0.552	0.555
Mean *n* ≈ 100	0.698	0.606	0.589	0.593
Number of outcomes				
Around 3	0.656	0.559	0.530	0.534
Around 5	0.704	0.626	0.610	0.614
Difference between categories				
$\gamma_{10}=$ 0.10	0.249	0.179	0.152	0.154
$\gamma_{10}=$ 0.30	0.820	0.695	0.662	0.667
$\gamma_{10}=$ 0.50	0.970	0.903	0.897	0.900
Distribution across categories				
Balanced	0.723	0.676	0.649	0.648
Unbalanced	0.707	0.647	0.621	0.623
Very unbalanced	0.610	0.454	0.440	0.451
Correlation bet. within ESs				
0.2–0.4	0.672	0.586	0.570	0.572
0.6–0.8	0.688	0.598	0.571	0.575
Total variance				
Small – 0.10	0.732	0.661	0.638	0.641
Large – 0.30	0.628	0.523	0.503	0.506

*Note:* LRT = likelihood ratio test; CE = correlated-effects models; RVE = Robust Variance Estimation method; CWB = Cluster wild bootstrapping.

Several anticipated trends are noted, including an increase in power with a greater number of studies, a larger difference between categories, reduced total variability, and a more balanced distribution of effect sizes across the moderator categories. Other factors, such as the correlation between outcome variables, did not appear to have a significant impact on power rates.

The power to detect an effect of an effect size-level variable was generally higher than the power to detect a study-level variable, often reaching relatively higher values across methods when the difference between categories was substantial (γ_1_ = 0.50). Among the methods, the three-level model that accounts for variability in the moderator effect across studies demonstrated the highest power rates. This was followed by the three-level model with RVE, which showed slightly greater power than both the correlated-effects models with RVE and with RVE and CWB approaches, while still adequately controlling for false positives.

Another important result is that RVE-based approaches were less affected by the uneven distribution of effect sizes across the moderator categories for effect size-level moderators (remember that when the characteristic was at the study level, the mean power for RVE-based methods was lower than 0.13, see Table 3). The ANOVA conducted on power rates revealed that the difference between categories was the most important factor (η^2^ = 0.63), with no other variables yielding substantial eta squared values (see Figure S6 in the Supplementary Material). The mean power disaggregated by each method and combination of conditions can be found in the Supplementary Material (*Effect size-level_Power.xlsx*).

## 6 Discussion

This study aimed to examine the Type I error rates and statistical power of methods available for conducting meta-regression with binary moderators in the presence of dependent effect sizes. In the following paragraphs, we summarize the key findings and provide practical recommendations for applied meta-analysts.

Regarding the moderator effect estimates, all methods generally performed well, although each showed a slight negative bias under certain conditions, specifically when the true effect was large and the effect sizes were very unbalanced distributed across the categories of the moderator. This latter condition (effect size distribution) also substantially reduced estimation efficiency (as reflected by the MSE), especially when the moderator corresponded to a study-level characteristic.

Perhaps the most relevant finding of this article is that using three-level models alone is not sufficient to ensure adequate control of Type I error, especially when the moderator variable represents a characteristic at the effect size level. Therefore, moving forward, if three-level models are used for meta-regression in meta-analyses, they should always be accompanied by RVE. This finding aligns with results reported by Fernández-Castilla and colleagues,^22^ where standard errors were significantly underestimated when a three-level model was applied without accounting for between-study variability in the moderator effect, which directly affected the Type I error. However, after the application of RVE, the Type I error was generally at the nominal level due to the small-sample corrections. This result is also consistent with the argument presented by Pustejovsky and Tipton^24^ that RVE should not be viewed as a distinct method for meta-analysis, but rather as “a technique for guarding against model mis-specification” (p. 427).

In this simulation study, we considered a scenario in which the moderator effect, defined by a within-study (effect size level) characteristic, varied across studies. This assumption helps explain why the traditional three-level model, which does not account for between-study variability in the moderator effect, produced inflated Type I error rates. In this mis-specified model, the between-study variance is likely underestimated because the variability in the moderator effect is not incorporated. This underestimated variance is then used to calculate the standard errors of the fixed effects, resulting in artificially small standard errors and, consequently, an increased risk of detecting false positives.

While the extent to which within-study moderator effects vary across studies is not well established, it is reasonable to expect such variability. Just as random-effects models are widely used based on the assumption that studies originate from different populations, there is little justification for assuming that the effect of a within-study moderator (e.g., the difference between two categories) remains constant across all studies. An alternative and more complex specification of the three-level model, which accounts for between-study variability in the moderator effect, is possible and appears to provide better control of Type I error. However, some inflation remains, particularly when the number of studies is small and effect sizes are evenly distributed across the moderator categories. This model also shows higher statistical power to detect an effect compared to RVE-based approaches. Unfortunately, this study did not explore whether applying RVE to this more complex model would have effectively controlled Type I error while preserving statistical power. Although Fernández-Castilla et al.^22^ showed that applying RVE to this type of model successfully reduced Type I error to nominal levels, further research is needed to assess its impact on power. If an applied researcher wishes to use this more complex three-level model, we recommend first conducting a likelihood ratio test to determine whether a model that allows the moderator effect to vary across studies provides a better fit than the traditional three-level meta-regression model.

The second most relevant result of this investigation is that, either if the moderator refers to a study or to an effect size characteristic, the method showing the best tradeoff between Type I error and power is the combination of three-level models and RVE. Therefore, this is the approach that we recommend when performing meta-regression with a categorical moderator, keeping in mind that when effect sizes are very unevenly distributed across the categories of the moderator, the power to detect an effect reduces substantially. In this regard, it is relevant to emphasize the importance of conducting power analyses prior to performing moderator analyses. Recently, a study developed power approximations for methods that account for the presence of dependent effect sizes within studies,^43^ ^,^ ^44^ extending those proposed by Hedges and Pigott.^7^ It is anticipated that power analyses for meta-regressions involving dependent effect sizes will soon be available, allowing meta-analysts to quantify in advance the probability of detecting the smallest effect size of interest.^45^

A third important result is that all RVE-based methods (correlated-effects models with RVE, and with RVE and CWB, and three-level models with RVE) effectively controlled Type I error across most combinations of conditions, regardless of whether the moderator pertained to a study or to an effect size-level characteristic. In contrast, three-level model approaches often led to inflated Type I error rates. Furthermore, when using RVE-based methods, the researcher does not need to worry about model specification (e.g., whether a moderator effect at the effect size level varies across studies). Additionally, the correlated-effects models with RVE and with RVE and CWB exhibited better convergence rates than multilevel and multivariate approaches, which aligns with the results observed by Park and Beretvas.^28^ However, this strong control of Type I error comes at the cost of power, which is lower for these methods compared to multilevel approaches. Additionally, correlated-effects models with CWB demonstrated higher power compared to the traditional application of RVE (with small-sample corrections), which is consistent with the findings of Joshi et al.^26^ However, the gain in power was relatively modest. Even so, it is important to note that Joshi et al.^26^ demonstrated that power gains with CWB are most significant for hypothesis tests involving multiple contrast hypotheses, that is, when analyzing categorical variables with more than two categories. Therefore, our results cannot be directly compared to those reported in their study.

A fourth finding is that the performance of these methods, in terms of Type I error and statistical power, varied depending on whether the moderator was a study-level or effect size-level characteristic. All methods that effectively controlled Type I error also demonstrated adequate power when the binary moderator referred to an effect size-level characteristic and the difference between categories was large. However, when the moderator represented a study-level characteristic, all methods showed lower power to detect an effect if the differences between overall effects across categories were small or moderate. Given that the average difference between categories is typically quite small (0.16, according to Cafri et al.^5^), our study illustrates how unlikely it is for any method to detect such a difference. This result should also caution researchers about using meta-regression models to analyze study-level variables under unfavorable conditions (e.g., small differences between categories, a small number of studies, or highly unbalanced distributions of effect sizes).

When the difference between the study-level moderator categories was large, all methods showed improved power, except when effect sizes were highly unbalanced across moderator categories. This finding is important, because such imbalances are common in meta-analyses. Our study demonstrates that, under these conditions, the probability of detecting a true moderator effect remains lower across all methods when the moderator is at the study level. This reduction in power, particularly when effect sizes are unevenly distributed across moderator categories, was also observed by Cuijpers et al.^6^ in simpler scenarios (i.e., when each study contributes only one effect size), and by Tipton^21^ for correlated-effects models with RVE and small-sample corrections. In this situation (i.e., high imbalance and study-level covariate), conducting power analyses becomes even more important, as performing meta-regression with any method may not be meaningful given the expected low statistical power.

Last, it is important to examine the inflated Type I error rates produced by the multivariate method when the moderator variable refers to an effect size-level characteristic. The main reason for this inflation is the same as that observed in the traditional three-level model: The specification of the multivariate model did not align with the data-generating process; specifically, the applied multivariate model fails to account for between-study variability in the moderator effect, which ultimately results in underestimated standard errors. Recent proposals for applying the multivariate method^37^ suggest including an additional random effect (i.e., fitting a multivariate three-level model) to account not only for the dependency among effect sizes arising from shared samples but also for the clustering of effect sizes within the same study. Incorporating this extra random effect would likely have improved the performance of the multivariate method, as well as the combination with the RVE, which was not examined in the present study.

Another interesting result is that the performance of three-level models was very similar either using Wald test or LRT, despite previous studies found that LRTs had more power to detect an effect for a continuous moderator.^9^

This simulation study is not without limitations. First, we examined only a subset of models commonly used in practice. Additional model specifications could have been considered, for instance, a multivariate three-level model, a version incorporating a random effect for the moderator, versions of both models combined with the RVE, or versions of both models combined with CWB. Second, we did not include a simulation condition in which the moderator effect was constant across studies or where the between-study variance differed by category (i.e., heteroscedastic variances). Last, unlike studies by Pustejovsky and Tipton^23^ or Joshi et al.,^26^ we did not explore more complex scenarios involving categorical moderators with more than two categories. Future research should investigate the power and Type I error properties of omnibus *F*-tests for comparing more than two groups, as well as the performance of multiple comparison corrections, such as Bonferroni or Tukey adjustments.

## Supporting information

10.1017/rsm.2026.10092.sm001 Fernández-Castilla et al. supplementary material Fernández-Castilla et al. supplementary material

## Data Availability

The code to run the simulation studies, the supplementary material, and some numerical results are available at https://osf.io/czwtp/overview.

## Footnotes

1
In multilevel models, when variables are left uncentered, the effects are not separated into their Level-1 and Level-2 components. However, when variables are centered around the cluster mean, the Level-1 effects represent only the within-cluster effect, independent of any Level-2 influence.

2
They did explore standard error bias estimates, which is directly related to Type I error.

3
It is important to note that, in practice, this model is often mis-specified because outcomes are typically obtained from the same sample within studies. Nevertheless, simulation studies show that the parameter estimates remain unbiased despite this mis-specification of the model.^18^ ^,^ ^19^

4
Note that in the field of biology, the Q1 sample size was way smaller (12). *N* = 35 is the average of the Q1 of multilevel meta-analysis in the medical field (Q1 = 26) and in psychology (Q1 = 50).

## Supplementary material

To view supplementary material for this article, please visit http://doi.org/10.1017/rsm.2026.10092.

## Long descriptions

### Table 1 Long description

The table is organized into four columns: Method/Parameter, Estimates (S E), p-Value, and Variance estimates.

1. Multivariate method: Intercept estimate is 0.374 (0.065) with sigma-hat sub u sub res squared equal to 0.330. Difference estimate is 0.413 (0.079) with a p-Value less than 0.001.

2. Three-level model: Intercept estimate is 0.518 (0.076). Difference estimate is 0.219 (0.133) with a p-Value of 0.099. Variance estimates are sigma-hat sub u sub res squared equal to 0.390 and sigma-hat sub w sub res squared equal to 0.080.

3. Three-level with additional random effect: Intercept estimate is 0.535 (0.074) with sigma-hat sub w sub res squared equal to 0.075. Difference estimate is 0.177 (0.161) with a p-Value of 0.271. Additional variance estimates are sigma-hat sub u sub res 0 squared (76) equal to 0.358 and sigma-hat sub u sub res 1 squared (26) equal to 0.550.

4. Three-level model with R V E: Intercept estimate is 0.518 (0.074) with sigma-hat sub u sub res squared equal to 0.390. Difference estimate is 0.219 (0.167) with a p-Value of 0.204 and sigma-hat sub w sub res squared equal to 0.080.

5. Correlated-effects models with R V E: Intercept estimate is 0.550 (0.076) with sigma-hat sub u sub res squared equal to 0.304. Difference estimate is 0.044 (0.152) with a p-Value of 0.772.

6. Correlated-effects models with R V E and C W B: Intercept and Difference estimates are not provided (marked with dashes), but the Difference p-Value is 0.782.

Navigate back to Table 1.

### Table 2 Long description

The table is organized under the main header Study-level moderator. The columns are divided into three main methodological categories: Multivariate (Wald test), Three-level model (Wald test and L R T), and R V E-based (Three-level with R V E, C E minus R V E, and C E minus C W B).

Data rows are organized by simulation conditions:

* Mean: Values range from 0.040 to 0.062.

* Number of studies: For k = 20, values are 0.067, 0.066, 0.067, 0.033, 0.033, and 0.035. For k = 40, values range from 0.046 to 0.058.

* Sample size: For Mean n is approximately 45, values range from 0.041 to 0.065. For Mean n is approximately 100, values range from 0.037 to 0.061.

* Number of outcomes: For Around 3, values range from 0.039 to 0.062. For Around 5, values range from 0.040 to 0.063.

* Distribution across categories: Balanced (0.049 to 0.061), Unbalanced (0.049 to 0.063), and Very unbalanced (0.021 to 0.064).

* Correlation between E S s: For rho = 0.4, values range from 0.040 to 0.063. For rho = 0.8, values range from 0.039 to 0.061.

* Total variance: Small - 0.10 (0.039 to 0.062) and Large - 0.30 (0.040 to 0.063).

Note: L R T is likelihood ratio test; C E is correlated-effects models; R V E is robust variance estimation; C W B is cluster wild bootstrapping. Bolded values indicate Type I errors outside the confidence interval.

Navigate back to Table 2.

### Figure 1 Long description

A multi-panel box plot titled Study-level Moderator. The Y-axis represents Type I error ranging from 0.000 to 0.075. Three horizontal red dashed lines are positioned at 0.0375, 0.050, and 0.0625. The X-axis lists six methods for each panel: Multivariate - Wald, Multilevel - Wald, Multilevel - L R T, Multilevel with R V E, C E - R V E, and C E - R V E and C W B.

* Balanced Panel: The first three methods (Wald and L R T) show median error rates slightly above 0.050. The latter three (R V E and C W B) show medians tightly clustered around 0.050.

* Unbalanced Panel: The first three methods show increased variability with medians rising toward 0.0625 and several high outliers. The R V E and C W B methods remain stable near 0.050.

* Very unbalanced Panel: The first three methods show medians above 0.0625. In contrast, the final three methods (Multilevel with R V E, C E - R V E, and C E - R V E and C W B) show a significant drop in Type I error, with medians falling below 0.025 and much wider interquartile ranges extending down toward 0.000.

Navigate back to Figure 1.

### Table 3 Long description

The table compares statistical power across several study-level moderator conditions. The columns are grouped into three main categories: Multivariate (Wald test), Three-level model (Wald test and L R T), and R V E-based (Three-level with R V E, C E minus R V E, and C E minus C W B).

Key data points by condition:

* Mean: Values range from 0.290 (Three-level with R V E) to 0.361 (L R T).

* Number of studies (k): Power increases from k equals 20 (range 0.204 to 0.297) to k equals 40 (range 0.374 to 0.427).

* Sample size: Mean n approximately 100 shows higher power (0.301 to 0.374) than mean n approximately 45 (0.278 to 0.349).

* Number of outcomes: Power is slightly higher for around 5 outcomes compared to around 3.

* Difference between categories (gamma sub 1 0): Power increases significantly as the difference grows from 0.10 (0.067 to 0.096) to 0.50 (0.526 to 0.640).

* Distribution across categories: Balanced distributions yield the highest power (0.412 to 0.439), while very unbalanced distributions show the lowest power, particularly for R V E-based methods (0.096 to 0.126).

* Correlation between E S s: Power is slightly higher at 0.4 correlation compared to 0.8.

* Total variance: Small variance (0.10) results in higher power (0.366 to 0.451) than large variance (0.30, range 0.214 to 0.273).

Note: L R T equals likelihood ratio test; C E equals correlated-effects models; R V E equals robust variance estimation; C W B equals cluster wild bootstrapping.

Navigate back to Table 3.

### Figure 2 Long description

The grid is organized with columns representing moderator magnitudes of 0.1, 0.3, and 0.5, and rows representing distribution balance: Balanced, Unbalanced, and Very unbalanced. The y-axis for all panels is Power, ranging from 0.0 to 1.0. The x-axis for each panel lists six methods: Multivariate - Wald, Multilevel - Wald, Multilevel - L R T, Multilevel with R V E, C E - R V E, and C E - R V E and C W B.

* Top Row (Balanced): Power increases significantly as magnitude moves from 0.1 (near 0.1 power) to 0.5 (near 0.8 power). All six methods perform similarly across magnitudes.

* Middle Row (Unbalanced): Power trends are similar to the balanced row but show a slight decrease in the R V E and C W B based methods compared to Wald and L R T methods at higher magnitudes.

* Bottom Row (Very unbalanced): Power is notably lower across all magnitudes compared to the balanced row. At a magnitude of 0.5, the Wald and L R T methods maintain power around 0.4, while the R V E and C W B methods drop significantly to approximately 0.1 to 0.2 power.

Across all panels, the first three methods (Multivariate - Wald, Multilevel - Wald, and Multilevel - L R T) consistently show higher power than the latter three (Multilevel with R V E, C E - R V E, and C E - R V E and C W B) as the degree of imbalance increases.

Navigate back to Figure 2.

### Table 4 Long description

The table compares Type I error rates for Multivariate Wald, Three-level model (Wald, L R T, and Wald random), and R V E-based methods (Three-level with R V E, C E minus R V E, and C E minus C W B).

Key data points by condition:

* Mean: Multivariate Wald (0.493), Three-level Wald (0.271), Three-level L R T (0.265), Three-level Wald random (0.084), Three-level with R V E (0.059), C E minus R V E (0.047), C E minus C W B (0.049).

* Number of studies: At k = 20, rates are slightly lower than at k = 40 for most methods. For example, Multivariate Wald is 0.487 at k = 20 versus 0.500 at k = 40.

* Sample size: Mean n approximately 100 shows higher error rates in Multivariate Wald (0.546) compared to n approximately 45 (0.440).

* Number of outcomes: Around 5 outcomes result in higher Multivariate Wald errors (0.528) than around 3 outcomes (0.458).

* Distribution: Balanced distributions generally show higher error rates in Multivariate Wald (0.522) compared to very unbalanced (0.455).

* Total variance: Large variance (0.30) significantly increases error rates across non-R V E methods, with Multivariate Wald reaching 0.579 compared to 0.407 for small variance (0.10).

Values for Multivariate Wald and Three-level Wald/L R T are consistently bolded, indicating they are outside the confidence interval for simulation error. R V E-based methods consistently maintain rates closer to the nominal 0.05 level.

Navigate back to Table 4.

### Table 5 Long description

The table is titled Mean power segmented by method and simulation condition. The columns are divided into Effect size-level moderator categories: Three-level model random Wald, and R V E-based methods which include Three-level with R V E, C E R V E, and C E C W B.

Data rows are as follows:

* Mean: Wald 0.680, Three-level with R V E 0.592, C E R V E 0.570, C E C W B 0.574.

* Number of studies: k = 20 (0.628, 0.517, 0.492, 0.497); k = 40 (0.731, 0.667, 0.648, 0.651).

* Sample size: Mean n approximately 45 (0.662, 0.578, 0.552, 0.555); Mean n approximately 100 (0.698, 0.606, 0.589, 0.593).

* Number of outcomes: Around 3 (0.656, 0.559, 0.530, 0.534); Around 5 (0.704, 0.626, 0.610, 0.614).

* Difference between categories: gamma sub 10 = 0.10 (0.249, 0.179, 0.152, 0.154); gamma sub 10 = 0.30 (0.820, 0.695, 0.662, 0.667); gamma sub 10 = 0.50 (0.970, 0.903, 0.897, 0.900).

* Distribution across categories: Balanced (0.723, 0.676, 0.649, 0.648); Unbalanced (0.707, 0.647, 0.621, 0.623); Very unbalanced (0.610, 0.454, 0.440, 0.451).

* Correlation between within E S s: 0.2 to 0.4 (0.672, 0.586, 0.570, 0.572); 0.6 to 0.8 (0.688, 0.598, 0.571, 0.575).

* Total variance: Small 0.10 (0.732, 0.661, 0.638, 0.641); Large 0.30 (0.628, 0.523, 0.503, 0.506).

Navigate back to Table 5.
