# Supplementary material for: Meta-regression with categorical moderators and dependent effect sizes: A simulation study
Source: Res Synth Methods. 2026 May 14;17(6):1234–60. doi: 10.1017/rsm.2026.10092 (PMC13732384; doi:10.1017/rsm.2026.10092)
Supplement: Supplementary file 1 [file S1759287926100921sup001.zip › S1759287926100921sup001/Supplementary material.pdf]

Supplementary material: Meta-Regression with Categorial Moderators and Dependent Effect Sizes: A Simulation Study

Page

Figure S1. *Bias of* γ_0 *when the moderator effect was at study-level……………* 2

Figure S2. *Bias of* γ_1 *when the moderator effect was at study-level……………* 3

Table S1. *Mean Squared Error by relevant conditions…………………………* 4

Figure S3. *Bias of* γ_0 *when the moderator effect was at effect size-level………* 5

Figure S4. *Bias of* γ_1 *when the moderator effect was at effect size-level………* 6

Figure S5. *Type I error distribution across methods, disaggregated by the degree of imbalance in the distribution of effect sizes across the moderator categories………………………………………………………………………………* 7

Figure S6. *Power distribution across methods, disaggregated by the degree of imbalance in the distribution of effect sizes across the moderator categories………………………………………………………………………………* 8

Figure S1.

Bias of γ_0 when the moderator effect was at study-level

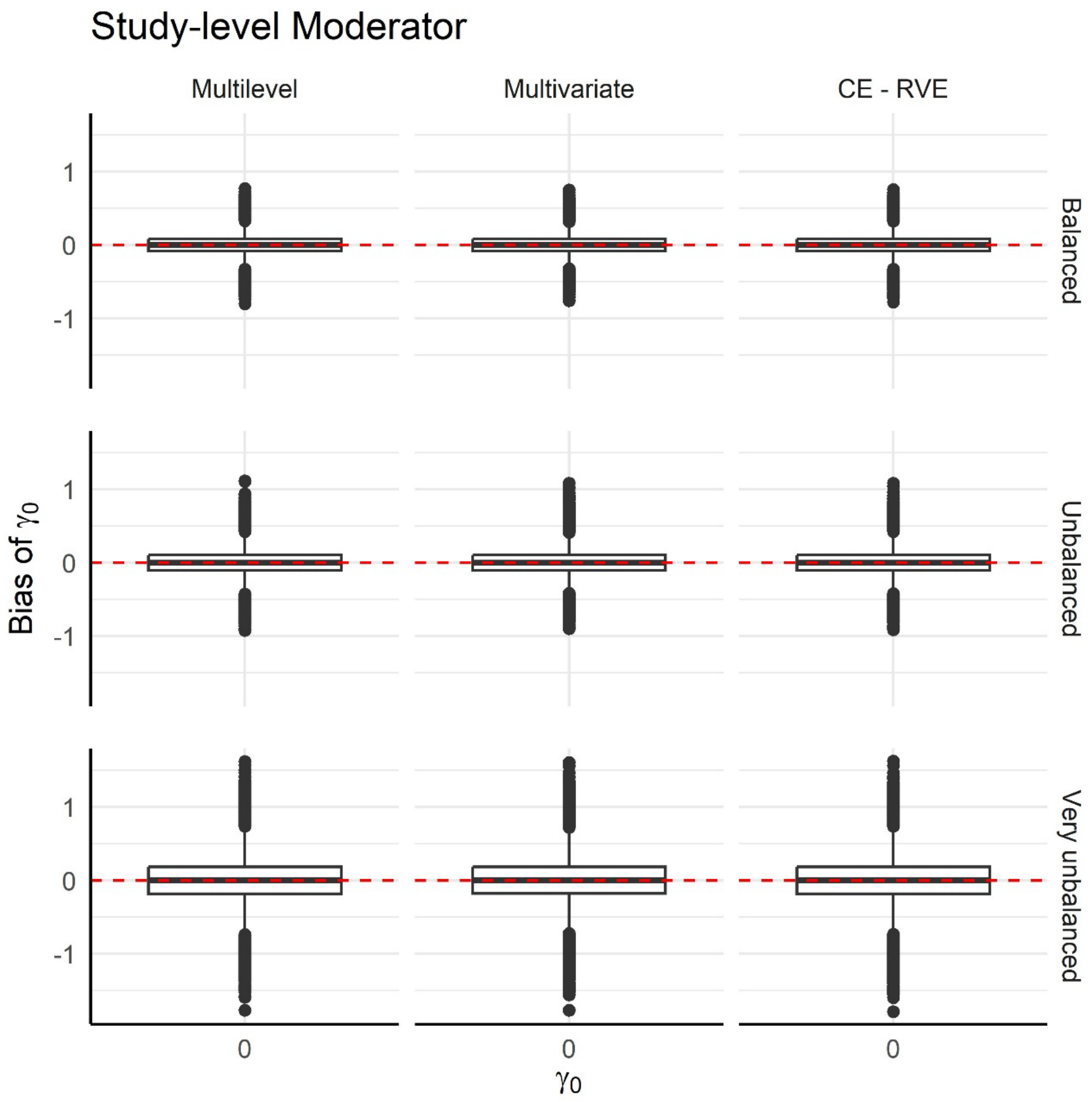

Notes. CE - RVE = correlated-effects model with robust variance estimation method.

Figure S2.

Bias of γ_1 when the moderator effect was at study-level

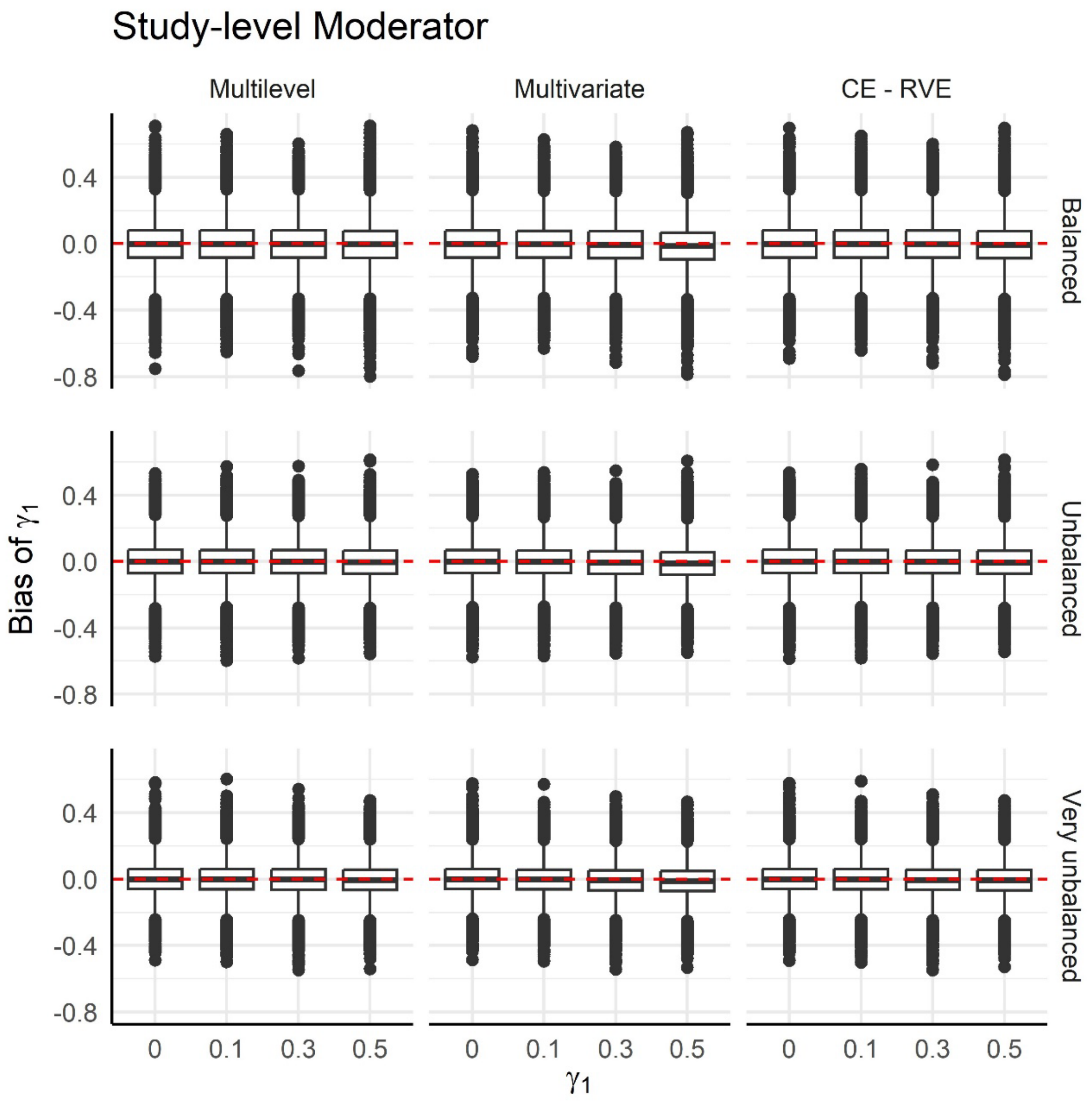

Notes. CE - RVE = correlated-effects model with robust variance estimation method.

Table S1.

Mean Squared Error by relevant conditions.

	Study-level moderator		Effect size-level moderator	
	γ_0	γ_1	γ_0	γ_1
**Studies**				
$k = 20$	.062	.018	.018	.008
$k = 40$	.031	.009	.009	.004
**Between studies variance**				
0.10	.027	.008	.008	.004
0.30	.065	.019	.018	.008
**Unbalanced degree**				
Balanced	.018	.018	.007	.007
Unbalanced	.030	.013	.009	.006
Very unbalanced	.090	.010	.024	.005

Figure S3

Bias of γ_0 when the moderator effect was at effect size-level

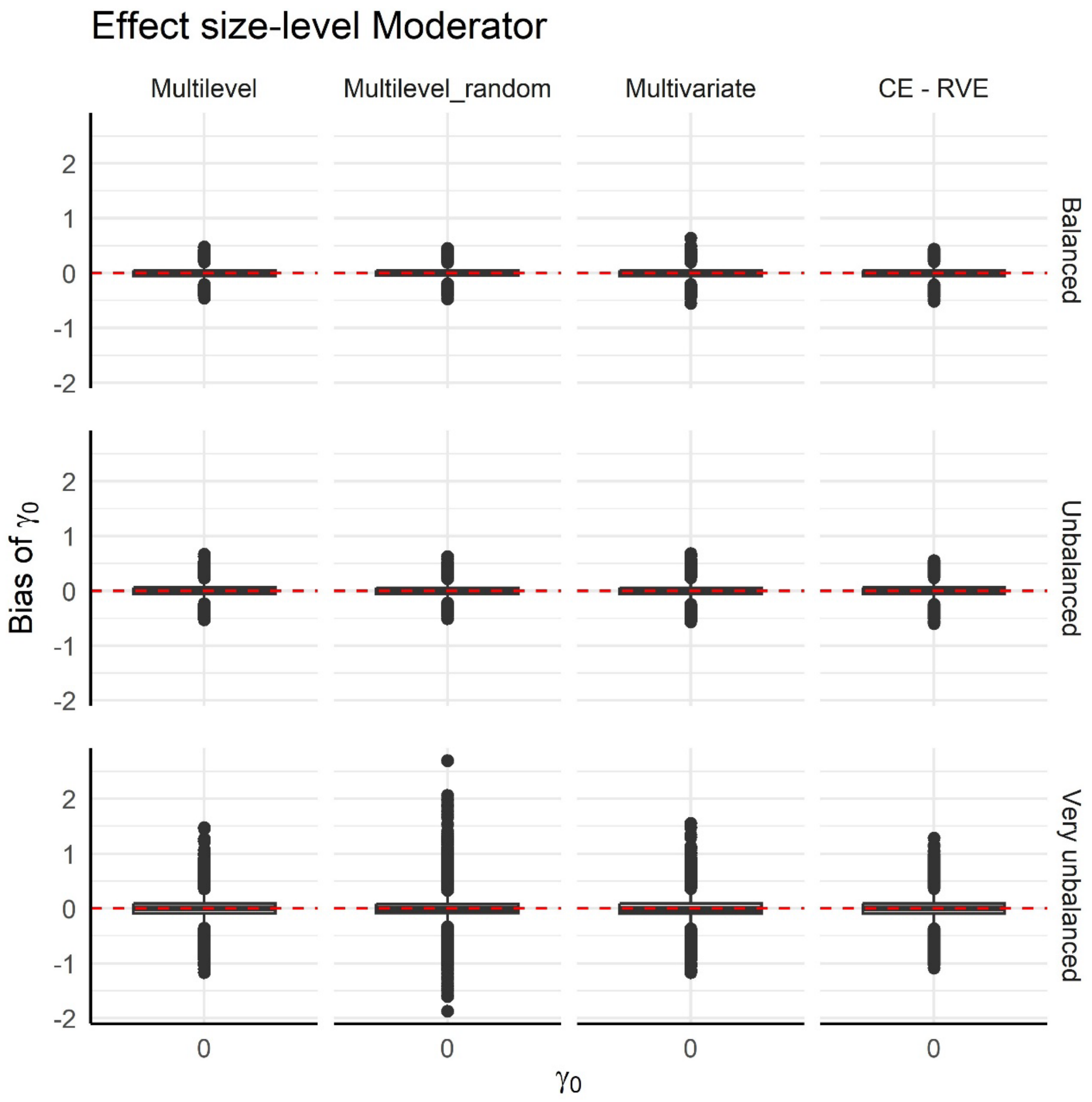

Notes. CE - RVE = correlated-effects model with robust variance estimation method.

Figure S4

Bias of γ_1 when the moderator effect was at effect size-level

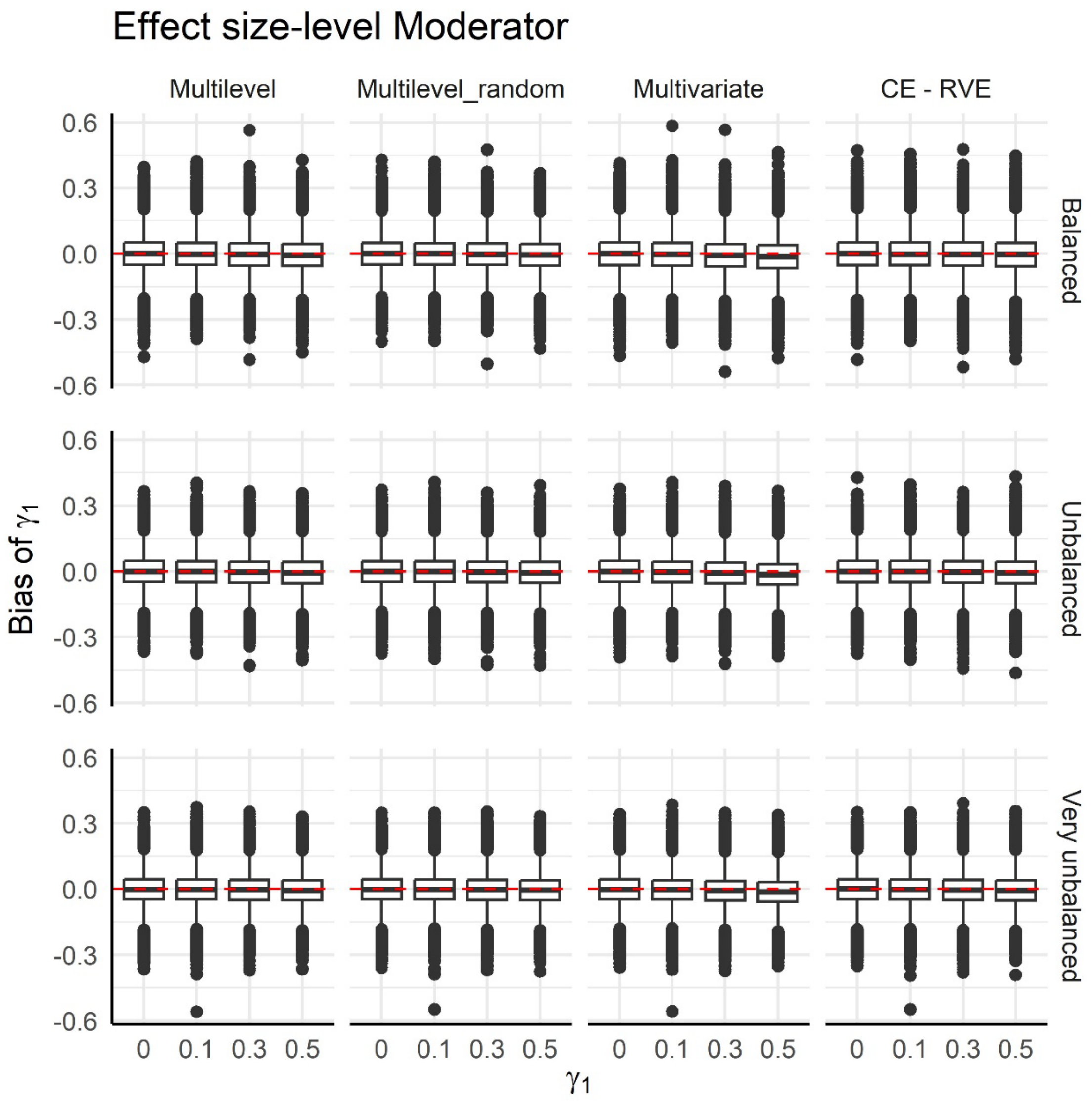

Notes. CE - RVE = correlated-effects model with robust variance estimation method.

Figure S5.

Type I error distribution across methods, disaggregated by the degree of imbalance in the distribution of effect sizes across the moderator categories

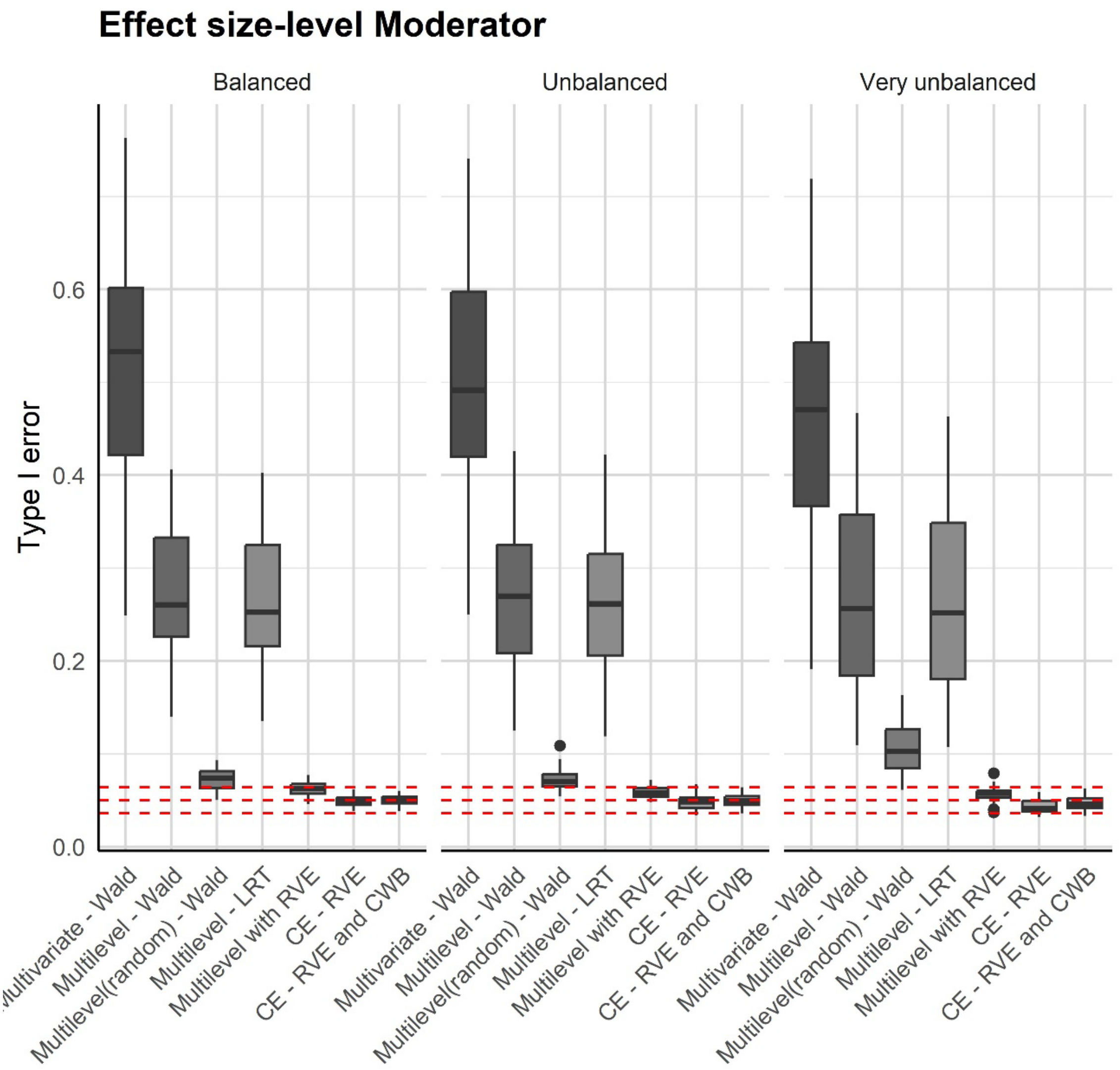

Notes. CE = correlated-effects model; RVE = Robust variance estimation method; CWB = Cluster wild bootstrapping.

Figure S6.

Power distribution across methods, disaggregated by the degree of imbalance in the distribution of effect sizes across the moderator categories

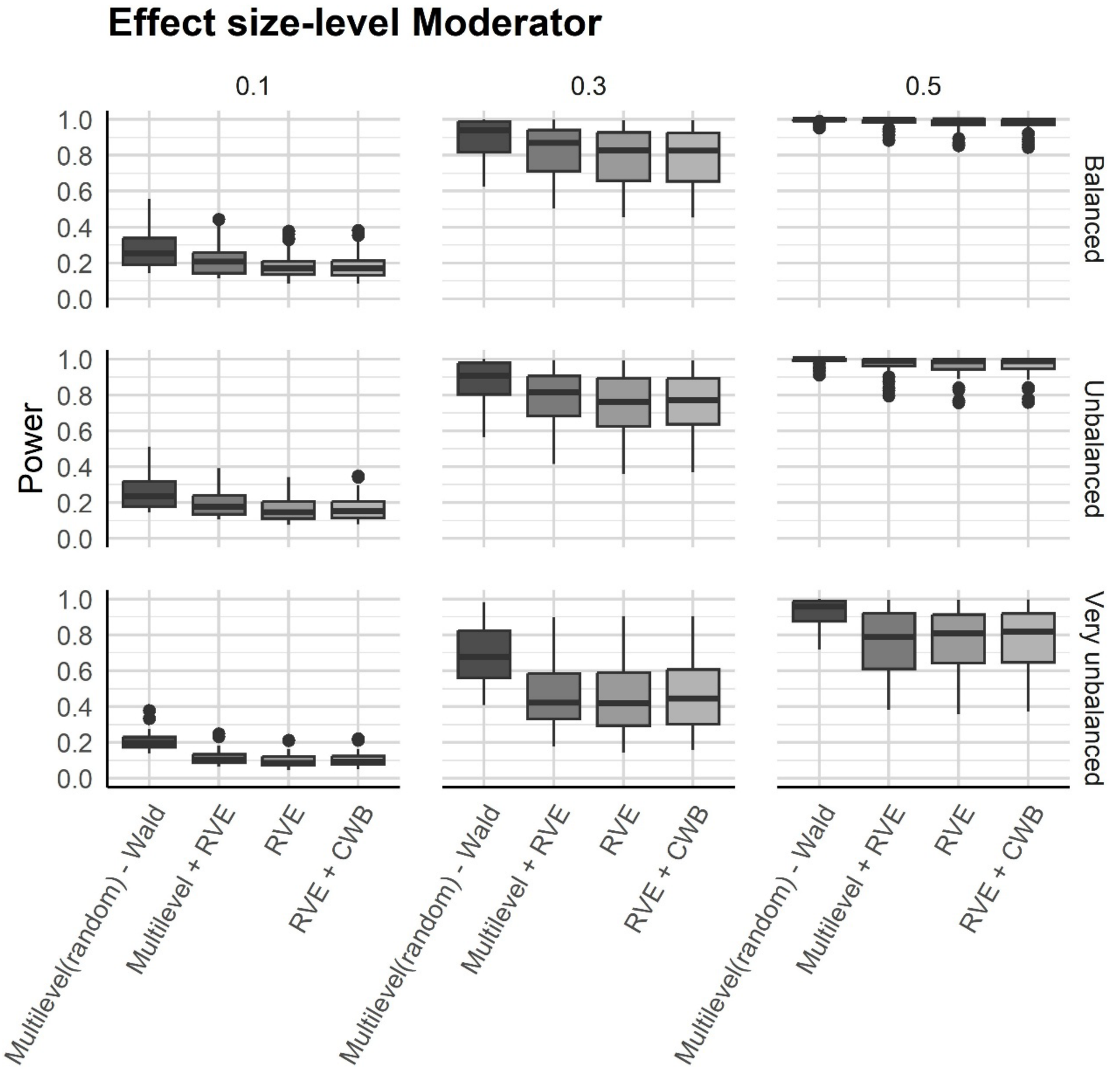

Notes. CE = correlated-effects model; RVE = Robust variance estimation method; CWB = Cluster wild bootstrapping.
